# Enhanced and prolonged baculovirus-mediated expression by incorporating recombinase system and *in cis* elements: a comparative study

**DOI:** 10.1093/nar/gkt442

**Published:** 2013-05-28

**Authors:** Li-Yu Sung, Chiu-Ling Chen, Shih-Yeh Lin, Shiaw-Min Hwang, Chia-Hsin Lu, Kuei-Chang Li, Alice S. Lan, Yu-Chen Hu

**Affiliations:** ^1^Department of Chemical Engineering, National Tsing Hua University, Hsinchu 300, Taiwan and ^2^Bioresource Collection and Research Center, Food Industry Research and Development Institute, Hsinchu 300, Taiwan

## Abstract

Baculovirus (BV) is a promising gene vector but mediates transient expression. To prolong the expression, we developed a binary system whereby the transgene in the substrate BV was excised by the recombinase (ΦC31o, Cre or FLPo) expressed by a second BV and recombined into smaller minicircle. The recombination efficiency was lower by ΦC31o (≈40–75%), but approached ≈90–95% by Cre and FLPo in various cell lines and stem cells [e.g. human adipose-derived stem cells (hASCs)]. Compared with FLPo, Cre exerted higher expression level and lower negative effects; thus, we incorporated additional *cis*-acting element [*oriP*/Epstein–Barr virus nuclear antigen 1 (EBNA1), scaffold/matrix attached region or human origin of replication (*ori*)] into the Cre-based BV system. In proliferating cells, only *oriP*/EBNA1 prolonged the transgene expression and maintained the episomal minicircles for 30 days without inadvertent integration, whereas BV genome was degraded in 10 days. When delivering *bmp2* or *vegf* genes, the efficient recombination/minicircle formation prolonged and enhanced the growth factor expression in hASCs. The prolonged bone morphogenetic protein 2 expression ameliorated the osteogenesis of hASCs, a stem cell with poor osteogenesis potential. Altogether, this BV vector exploiting Cre-mediated recombination and *oriP*/EBNA1 conferred remarkably high recombination efficiency, which prolonged and enhanced the transgene expression in dividing and non-dividing cells, thereby broadening the applications of BV.

## INTRODUCTION

Baculovirus (BV) is a dsDNA virus that naturally infects insects, but it also efficiently transduces various mammalian cell lines and stem cells such as induced pluripotent stem (iPS) cells (1) and embryonic stem cells (2). BV is non-pathogenic to humans and is easy for genetic manipulation, making it amenable to handle and produce in biosafety level 1 facilities (3). These features have prompted the use of BV vectors for *in vitro* and *in vivo* gene delivery (4,5), vaccine development (6) and assay development (7). Additionally, BV transduces bone marrow-derived mesenchymal stem cells (BMSCs) (8,9) and adipose-derived stem cells (ASCs) (10) at efficiencies >95% without obstructing cell differentiation (11). Transduction of BMSCs derived from human (hBMSCs) and rabbit (rBMSCs) with a recombinant BV expressing the potent osteogenic growth factor bone morphogenetic protein 2 (BMP2) accelerates osteogenesis *in vitro*, and transplantation of the cells into animals augments bone healing *in vivo* (9,12,13), thus implicating the potential of BV for regenerative medicine.

However, one drawback of BV is that it typically enables transient expression for <7 days (14–16) unless antibiotic selection (17) or vector engineering for transgene integration is adopted (2,18,19), which may hinder its direct applications in some scenarios. For instance, ASCs have become a popular cell source for bone engineering, but ASCs are poorer than BMSCs in osteogenesis potential (10). Consequently, transplantation of BV-transduced rabbit ASCs (rASCs) that transiently express BMP2 and vascular endothelial growth factor (VEGF) only results in subpar bone healing in rabbits (10). As such, prolonged BV-mediated BMP2/VEGF expression may ameliorate the ASCs osteogenesis and promote bone healing. The BV-mediated expression duration is short mainly due to the non-replicating nature of the circular BV genome and the viral genome degradation within mammalian cells. Therefore, we hypothesized that shedding the cargo from the BV genome to form an extrachromosomal minicircle may prolong the transgene expression.

Site-specific recombinases (e.g. ΦC31, Cre and FLP) are enzymes that catalyze DNA exchange between short target sequences (20), leading to excision of intervening DNA and subsequent recombination as a circular molecule (DNA minicircle) if the flanking target sites are in direct orientation (21). ΦC31 mediates excision/recombination between the heterotypic sites *attP* and *attB*, whereas Cre and FLP catalyze excision/recombination events between two identical sites (loxP for Cre and Frt for FLP) (21). These recombinases are widely used for gene integration, excision (22) or activation and are used as molecular switches to temporally and spatially control gene expression (21). However, their application in prolonging transgene expression is rare. Furthermore, the recombination efficiencies of ΦC31 and FLP are low; hence, codon-optimized ΦC31 (ΦC31o) and FLP (FLPo) were recently developed (23).

To prolong the BV-mediated expression, we attempted to split the transgene cassette as a minicircle. We constructed the BV vectors expressing ΦC31o, Cre or FLPo and the hybrid substrate BV vector and compared their efficiencies for recombination/minicircle formation in different mammalian cells. Besides, o*riP* derived from Epstein–Barr virus is an origin of replication (*ori*) that interacts with Epstein–Barr virus nuclear antigen 1 (EBNA1) to coordinate the replication of *oriP*-containing episomes (24). Scaffold/matrix attached region (S/MAR) and human lamin B2 *ori* are DNA elements that support episome maintenance (25) and chromosome replication (26). To extend the existence of minicircles and transgene expression in proliferating cells, we further incorporated these *cis*-acting elements into the hybrid BV and evaluated the persistence of transgene expression, minicircles and BV genome. Whether the hybrid BV system was applicable to non-proliferating human ASCs (hASCs) to extend growth factor expression and promote osteogenesis was also explored.

## MATERIALS AND METHODS

### Cells, recombinant BV preparation and transduction

Mammalian cell lines HeLa, HEK293, Huh-7, baby hamster kidney and rhabdomyosarcoma were cultured using Dulbecco’s modified Eagle’s medium (DMEM) medium. New Zealand White rabbit ASCs (rASCs) and BMSCs (rBMSCs) were isolated as described (10,12). Human ASCs (hASCs) were obtained from the Food industry Research and Development Institute (Hsinchu, Taiwan). Culture of these cell lines and stem cells are described in details in Supplementary Materials and Methods.

To prepare recombinant BV constructs, the exogenous cassette was first cloned into a donor plasmid, followed by virus generation using the Bac-To-Bac™ system (Invitrogen, Carlsbad, CA). All primers used for virus construction are listed in Supplementary Table S1. To generate recombinase-based donor plasmids (pBacΦC31o, pBacCre and pBacFLPo), the cDNAs of ΦC31o, Cre and FLPo were PCR-amplified from pPGKPhiC31obpA, pPGKCrebpA and pPGKFLPobpA (Addgene, Cambridge, MA), respectively, and subcloned into pBacCMV5 (27) using EcoRI/NotI. The construction of pBacALF was divided into three steps. First, the DNA fragment encoding the tandem recombination sites (XhoI-attP-loxP-Frt-BamHI-StuI-attB-loxP-Frt-HindIII) was chemically synthesized (full sequence is listed in Supplementary Materials and Methods) and subcloned into pFastBac Dual (Invitrogen) by XhoI/HindIII digestion to yield pALF. Second, the *d2egfp*-SV40pA fragment was PCR amplified from pd2EGFP-N1 (Clontech) and inserted into the BamHI site of pALF to form pALF-dE. Finally, the CMV promoter (flanked by StuI/SmaI) was PCR-amplified from pcDNA3.1(+) (Invitrogen) and inserted into the *Stu*I site of pALF-dE to yield pBacALF (a StuI site was still available in between the SV40pA and CMV promoter). To generate other three pBacALF-based donor plasmids (pBacALF-5k, pBacALF-10k and pBacALF-15k), stuffer DNA of different sizes (≈3, 8, 13 kb) were obtained from EcoRV-digested *Escherichia coli* genome and subcloned into the StuI site of pBacALF. Additionally, pBacALF-CdE (Supplementary Figure S1) was constructed as a standard for absolute quantitative real-time PCR (qPCR) analysis.

The construction of pBacCdE/W was divided into 2 steps. First, a woodchuck hepatitis virus post-transcriptional regulatory element (WPRE) was PCR-amplified and subcloned into pBacCMV5 with HindIII to form pBacCMV-WPRE. Second, *d2egfp* was SalI/NotI-digested from pd2EGFP-N1 (Invitrogen) and subcloned into pBacCMV-WPRE to form pBacCdE/W. To further add two loxP sites outside the transgene cassette, two loxP fragments (34 bp) flanked by SphI/NheI or NotI/PstI were synthesized using two sets of primers (Table S1) and were separately inserted into pBacCMV5 to form pBacL-CMV. Next, the *d2egfp*-WPRE-SV40pA fragment was PCR-amplified from pBacCdE/W and inserted into BamHI site of pBacL-CMV to form pBacL-CdE/W. To generate pBacL-CdE/W-CEO, the fragment of EBNA1-*oriP* was EcoRI/SalI-digested from pREP4 (Invitrogen) and inserted into pBacL-CdE/W. The CMV promoter was subsequently subcloned into the EcoRI site to complete the construction of pBacL-CdE/W-CEO. The map and sequence of the plasmid are described in details in Supplementary Materials and Methods. The construction of pBacL-CdE/WS involved three steps. First, S/MAR fragment was PCR-amplified from pEPI-eGFP (PlasmidFactory, Bielefeld, Germany) and subcloned into pBacCMV-WPRE with AvrII to form pBacC-WS. Second, the WPRE-S/MAR fragment was PCR-amplified and subcloned into pd2EGFP-N1 with NotI to form pdE/WS. Finally, the full cassette of *d2egfp*-WPRE-S/MAR-SV40pA was PCR-amplified and subcloned into pBacL-CMV using BamHI/StuI to complete the construction of pBacL-CdE/WS. To generate pBacL-CdE/W-hO, Lamin B2 *ori* was directly PCR-amplified from HEK293 genome and subcloned into pBacL-CdE/W using EcoRI.

To generate pBacL-CB/W-CEO and pBacL-CV/W-CEO, the CMV-*bmp2* and CMV-*vegf165* cassettes were first digested from pBacCB (28) and pBacCV (12) with EcoRI/XhoI and SalI/NotI, respectively, and subcloned into pBacL-CMV to form pBacL-CB and pBacL-CV. The WPRE-SV40pA-CMV-EBNA1-*oriP* fragment was digested from pBacL-CdE/W-CEO with NotI and inserted into pBacL-CB and pBacL-CV to complete the constructs. The recombinant BV vectors were amplified by infecting Sf-9 insect cells, titrated by end-point dilution method and stored as described previously (29).

For transduction, mammalian cells cultured in 6-well plates overnight were washed once with phosphate buffered saline, and transduced by adding 500 µl solution comprising 100 µl unconcentrated BV and 400 µl sodium bicarbonate-deficient DMEM containing 10% fetal bovine serum (FBS; HyClone, Rockford, IL). After incubation at room temperature on the shaker for 6 h, virus solution was replaced by fresh medium containing 3 mM sodium butyrate (Sigma). The transduced cells were incubated for 24 h and then cultured in complete medium. For the induction of osteogenesis, hASCs were cultured in DMEM (high glucose) medium containing 10% FBS, 10 mM β-glycerol phosphate (Sigma), 100 nM dexamethason (Sigma) and 150 µM ascorbic acid (Sigma).

### Transgene expression and viability measurements

The percentage of GFP+ cells (%GFP+ cells) and mean fluorescence intensity (FI) were measured using a flow cytometer (FACSCalibur, Becton Dickinson, Franklin Lakes, NJ). The values were calculated by measuring each sample three times and counting 10,000 cells in each measurement. The BMP2 and VEGF levels in the medium were measured using enzyme-linked immunosorbent assay (ELISA) kits specific for human BMP2 and VEGF (R&D Systems, Minneapolis, MN). The cell viabilities were measured using MTT (3-(4,5-dmethylthiazol-2-yl)-2,5-diphenyltetrazolium bromide) assay as described (16).

### qPCR

The qPCR reactions were conducted using StepOnePlus Real-Time PCR Systems (Applied Biosystems, Foster City, California) with 5 µl of sample (200 ng), 2.5 µl of forward and reverse primers (4 µM) and 10 µl of SYBR Green PCR Master Mix (Applied Biosystems). To quantify the absolute episome copy number, DNA samples were extracted from the transduced cells, and two different primer pairs were designed to separately detect *d2egfp* and minicircle copy number (Supplementary Table S2 and Supplementary Figure S1). The pBacALF-CdE plasmid (Supplementary Figure S1) was serially diluted (200, 20, 2, 0.2 and 0.02 pg) and quantified by qPCR to generate the standard curve. The absolute *d2egfp* and minicircle copy numbers (copies/10^6^ cells) were then quantified based on 200 ng of each sample on the assumption of 6.8 pg of DNA per cell (30).

For relative quantifications, two primer pairs (Supplementary Table S2 and Supplementary Figure S2) targeting *gp64* (for BV copy number) and the minicircle were used for qPCR reactions and *gapdh* served as the internal control. All data were normalized to those at 1 day post-transduction (dpt).

### Fluorescence *in situ* hybridization

To prepare the *d2egfp* probe, the *d2egfp* fragment was digested from pd2EGFP-N1 with *SalI*/*NotI*, purified and labeled with fluorescein dUTP using the Random Primed DNA Labeling Kit (Roche, Indianapolis, IN). HEK293 cells seeded on T25 flasks (2 × 10^6^ cells) were transduced with the hybrid vector. At 5 dpt, the mitotic HEK293 cells were treated with colcemid (10 µg/ml, Sigma) for 2 h. After centrifugation, the mitotic cells were incubated in the hypotonic KCl solution (0.075 M) at 37°C for 20 min and fixed in methanol/acetic acid (3:1). After fixation, the cells were dropped onto slides and dehydrated at 60°C overnight. Subsequent hybridization and 4′,6-diamidino-2-phenylindole (DAPI) staining were performed as described previously (11). The probe signals were observed using a confocal microscope (Nikon TE2000, Nikon, Tokyo, Japan).

### Real-time quantitative reverse transcription PCR

Real-time quantitative reverse transcription PCR (qRT-PCR) was performed to quantify the transcription levels of osteogenic marker genes (Runx2, alkaline phosphatase, osteopontin and osteocalcin). Total cellular RNA was extracted from hASCs with the NucleoSpin® RNA II Kit (Macherey-Nagel, Duren, Germany), and 1 µg of the RNA was reverse transcribed to cDNA with the Omniscript RT Kit (Qiagen, Hilden, Germany). Five microliter of the diluted cDNA (500×) was used for qPCR reactions, using primer sets specific for the osteogenic marker genes (Supplementary Table S3). All data were normalized against those at 1 dpt.

### Alizarin red staining

For Alizarin red staining detection of mineralization, transduced hASCs were cultured in osteoinduction medium for 7 or 14 days, washed with phosphate buffered saline, fixed with 10% formalin (Sigma) for 10 min, washed again with deionized water, followed by treatment with Alizarin red solution (40 mM (pH 4.2), Sigma).

## RESULTS

### Recombinase-based hybrid BV vectors and efficiency of DNA minicircle formation

**Figure 1.**
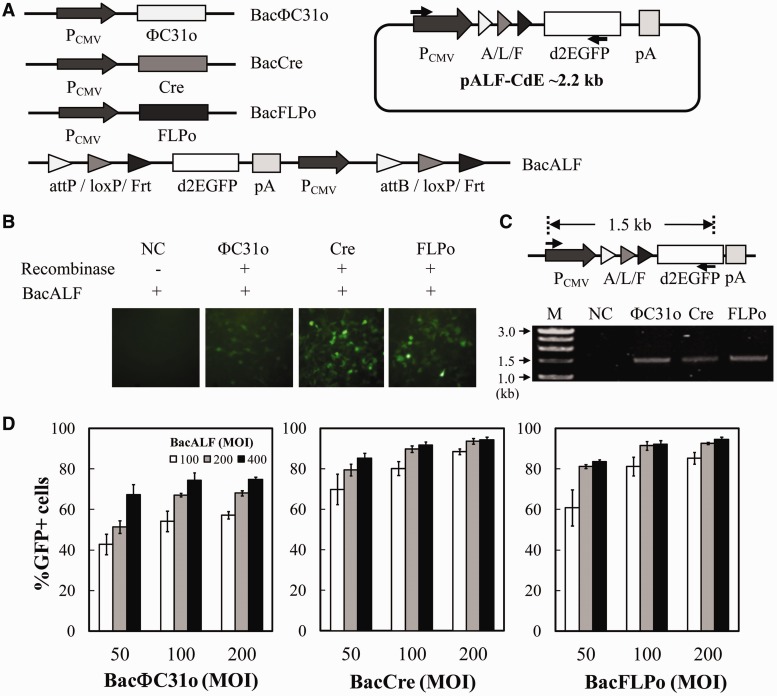
Recombinase-based BV constructs and confirmation of minicircle formation. (**A**) Schematic illustration of BacΦC31o, BacCre, BacFLPo and BacALF containing the synthetic tandem recognition sites (attP/loxP/Frt and attB/loxP/Frt). After successful recombination, a ≈2.2 kb minicircle (pALF-CdE) was formed. A/L/F indicates the recombined tandem recognition sites. P_CMV_, cytomegalovirus immediate-early promoter, pA, polyadenylation signal. (**B**) Confirmation of recombination by d2EGFP expression. (**C**) Confirmation of recombination by PCR. HEK293 cells were co-transduced with BacALF (MOI 100), and the corresponding recombinase-encoding BV (MOI 100), or singly transduced with BacALF as negative control (NC). At 1 dpt, d2EGFP expression was observed (200×) and intracellular minicircle formation was verified by PCR assay using the primers spanning a 1.5 kb region in the recombined minicircle. The arrows indicate the primer binding sites. (**D**) Dependency of recombination efficiencies on MOI. HEK293 cells were co-transduced with BacALF and the recombinase-expressing BV at various MOI combinations and were subjected to flow cytometry at 1 dpt.

We first constructed three BV vectors (BacΦC31o, BacCre and BacFLPo, Figure 1A) each expressing the respective recombinase, and a substrate BV vector BacALF (Figure 1A), which harbored *d2egfp* (encoding destabilized d2EGFP) flanked by tandem recombination sites (attP-loxP-Frt and attB-loxP-Frt). After co-transduction with BacALF and the recombinase-expressing BV, this design theoretically enabled recombinase-mediated transgene excision and recombination to form a ≈2.2 kb minicircle (pALF-CdE, Figure 1A), resulting in the placement of *d2egfp* downstream of CMV promoter. Therefore, d2EGFP expression indicated the minicircle formation. Indeed, HEK293 cells expressed no d2EGFP at 1 dpt when singly transduced with BacALF (negative control) but expressed d2EGFP on co-transduction (Figure 1B), indicating the requirement of recombinase for d2EGFP expression. PCR analysis (Figure 1C) using the primers spanning a 1.5 kb region solely in the recombined pALF-CdE further revealed the generation of a 1.5 kb fragment from the co-transduced cells, thereby attesting the minicircle formation.

The dependence of recombination efficiency on virus dosage was examined by co-transducing HEK293 cells at various multiplicity of infection (MOI) combinations. The flow cytometry, which measured the percentage of cells emitting fluorescence (%GFP+ cells) at 1 dpt (Figure 1D) and hence the recombination efficiency, revealed that BacΦC31o yielded the lowest recombination efficiency (≈40–75%), whereas BacCre and BacFLPo conferred higher recombination efficiencies in the range of ≈70–95% and ≈60–95%, respectively. When the BacALF MOI was 200–400, BacCre and BacFLPo at MOI 100–200 conferred statistically similar recombination efficiencies (≈90–95%, *P* > 0.05); thus, BacCre and BacFLPo were selected (MOI 100) for co-transduction with the substrate vector (MOI 200) in ensuing experiments.

### Effects of insert size on the formation of minicircle

**Figure 2.**
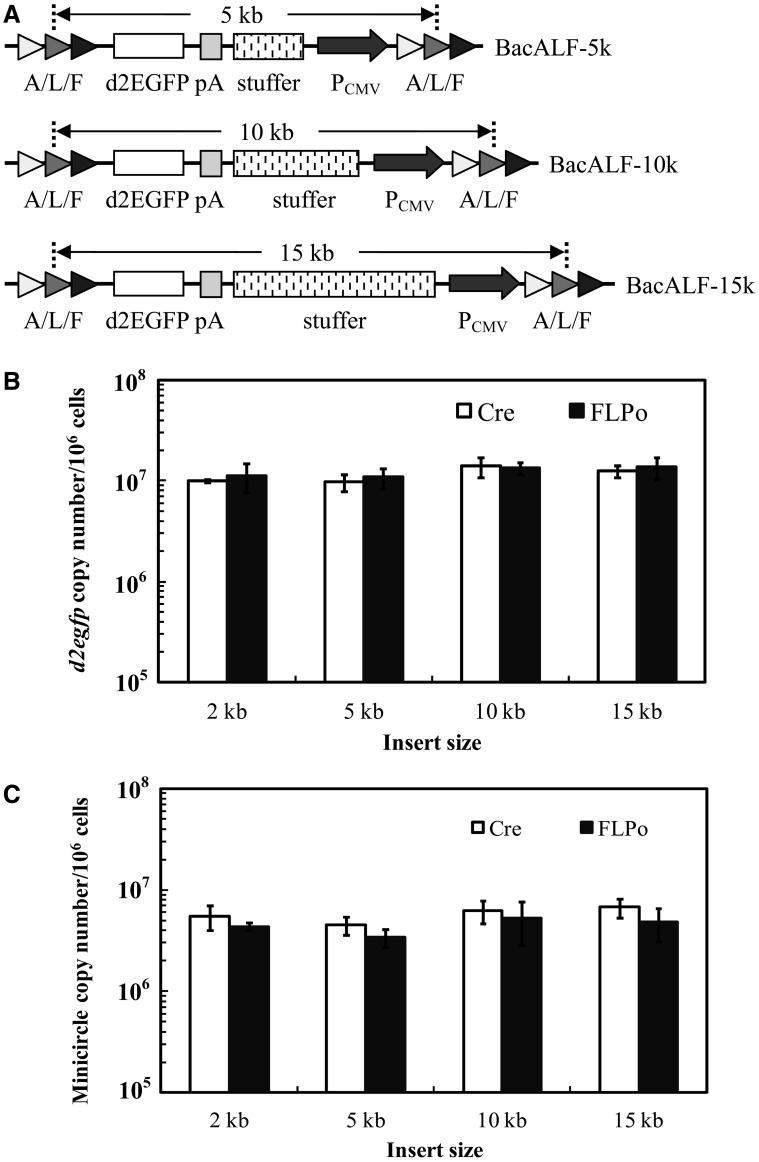
Effects of insert size on recombination and minicircle formation. (**A**) Substrate BV constructs carrying stuffer DNA of different sizes. (**B**) *d2egfp* copy numbers. (**C**) Minicircle copy numbers. HEK293 cells were co-transduced with BacCre (or BacFLPo, MOI 100) and the substrate BV (BacALF, BacALF-5k, BacALF-10k or BacALF-15k) at MOI 200. At 1 dpt, the absolute copy numbers of *d2egfp* and minicircles were analyzed by qPCR using *d2egfp-* and minicircle-specific primers, respectively. The insert sizes were actually 2.2, 5.3, 10.4 and 15.4 kb, respectively, but are shown as 2, 5, 10 and 15 kb for simpler presentation.

The insert size in BacALF and the resultant pALF-CdE size was ≈2.2 kb. To assess how the insert size, and hence the distance between the 2 flanking recognition sites, influenced the minicircle formation, we constructed BacALF-5k, BacALF-10k and BacALF-15k (Figure 2A), which resembled BacALF but carried an additional bacterial stuffer DNA (≈3, 8 or 13 kb) such that the overall insert size reached ≈5, 10 and 15 kb, respectively. One day after co-transducing HEK293 cells with BacCre or BacFLPo and the substrate BV, the copy numbers of intracellular *d2egfp* (Figure 2B) and minicircle (Figure 2C) were quantified by absolute qPCR. Figure 2B delineates statistically similar (*P* > 0.05) *d2egfp* copy numbers (≈9.8–14.0 × 10^6^ copies/10^6^ cells), regardless of the insert size or recombinase, thus proving that all substrate BV vectors delivered comparable amounts of transgenes into the cells. The copy numbers of minicircles (≈3.3–6.7 × 10^6^ copies/10^6^ cells) were also independent of the recombinase and insert size, demonstrating that BacCre and BacFLPo mediated the recombination/minicircle formation at similar efficiencies. As the total *d2egfp* copy number was the sum of BV genome and minicircle, by calculating the number ratios of minicircle to *d2egfp* we estimated that ≈35–50 copies of minicircles were formed from every 100 copies of BV genome.

### Recombination efficiency and viability in different mammalian cells

**Figure 3.**
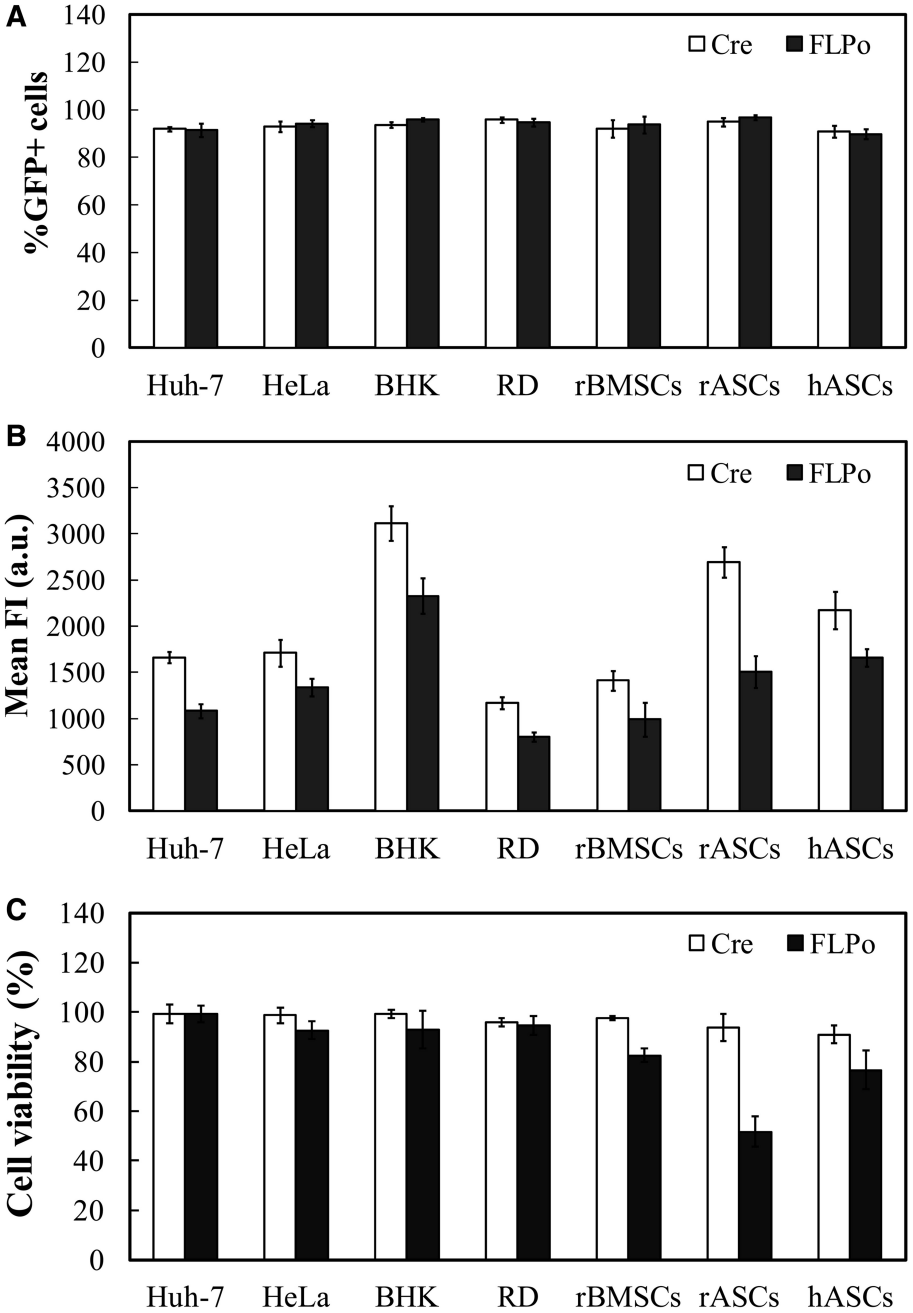
Recombination efficiencies and cytotoxicities in different cells. (**A**) Recombination efficiency. (**B**) Mean FI. Cells were co-transduced with BacCre (or BacFLPo, MOI 100) and BacALF (MOI 200). At 1 dpt, %GFP+ cells and mean FI were analyzed by flow cytometry. The mean FI values are expressed as arbitrary units (a.u.). (**C**) Relative cell viability. Cells were singly transduced with BacCre or BacFLPo at MOI 100, and the cytotoxicities were measured by MTT assay at 2 dpt.

To examine whether the recombination occurred in cells from different tissues and species, we co-transduced Huh-7 (human hepatoma), HeLa (human cervical carcinoma), baby hamster kidney, human rhabdomyosarcoma, rBMSCs, rASCs and hASCs with BacCre + BacALF or BacFLPo + BacALF. The fluorescence microscopy (Supplementary Figure S3) and flow cytometry (Figure 3A) demonstrated that both BacCre and BacFLPo resulted in recombination in all these cells at comparable efficiencies (≈90–96%). However, BacFLPo yielded lower mean FI than BacCre (Figure 3B) and led to lower viability in rBMSCs, rASCs and hASCs (Figure 3C). In contrast, BacCre conferred higher expression level and exerted little influence on viability in these cells (Figure 3C). Therefore, Cre/loxP system was chosen for subsequent vector construction.

### Comparison of *cis*-acting elements for prolonged expression and minicircle maintenance

**Figure 4.**
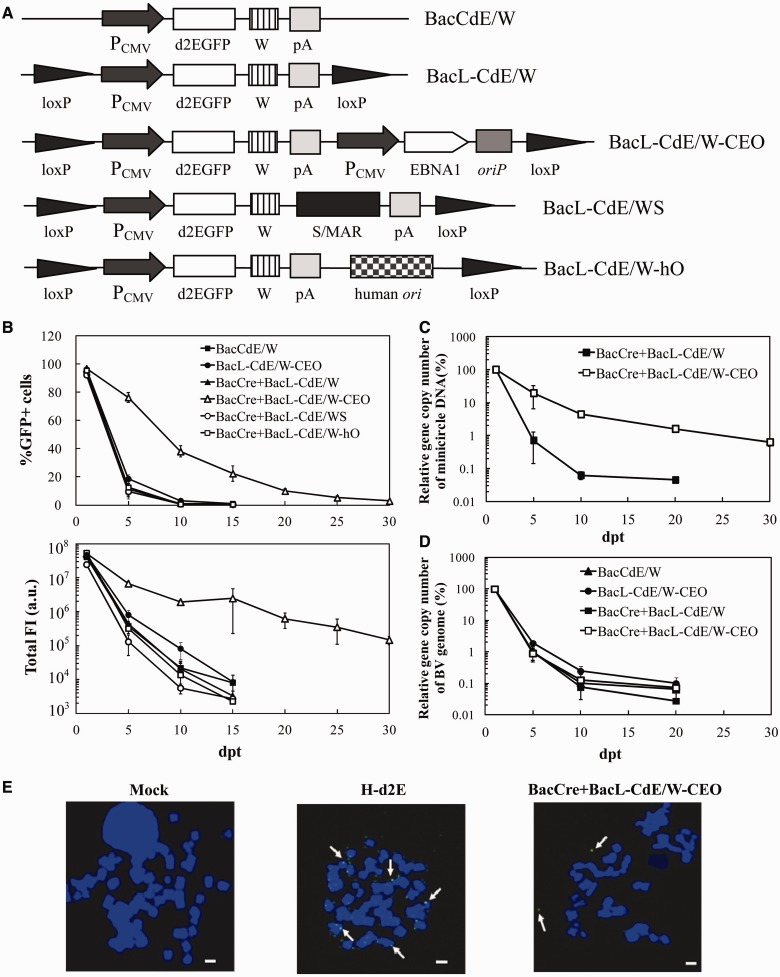
Comparison of *cis*-acting elements for prolonged expression and minicircle maintenance. (**A**) Substrate BV constructs harboring different *cis*-acting elements. W, WPRE sequence. *oriP*/EBNA1 was derived from Epstein–Barr virus; S/MAR element was derived from the 5′ end of human interferon β gene; human *ori* was derived from 3′ end of the lamin B2 gene. (**B**) Duration of d2EGFP expression as measured by flow cytometry. (**C**) Relative minicircle copy numbers. (**D**) Relative BV genome copy numbers. HEK293 cells were either singly transduced with BacCdE/W (MOI 200) or BacL-CdE/W-CEO (MOI 200), or co-transduced with BacCre (MOI 100) and the substrate BV (MOI 200). The copy numbers were determined by qPCR using primers specific to the minicircle or BV genome and normalized against those at 1 dpt. (**E**) Examination of transgene integration by FISH. HEK293 cells were co-transduced with BacCre and BacL-CdE/W-CEO and subjected to FISH at 5 dpt using the *d2egfp*-specific probe. Mock-transduced cells served as negative control, whereas H-d2E cells (HEK293 cells harboring the integrated *d2egfp*) served as positive control. The images are representative of 100 metaphases. Scale bar, 1 µm.

We next sought to extend the transgene expression by appending *cis*-acting elements to the minicircle. BacCdE/W is a BV vector (10) wherein d2EGFP was directly driven by CMV promoter. Based on BacCdE/W, we first constructed BacL-CdE/W, which contained two flanking loxP sites (Figure 4A), and then developed three more BacL-CdE/W-based substrate vectors (Figure 4A): BacL-CdE/W-CEO harbored the *oriP*/EBNA1 element; BacL-CdE/WS carried S/MAR downstream of *d2egfp*, whereas BacL-CdE/W-hO accommodated a human lamin B2 *ori*. HEK293 cells were singly transduced with BacCdE/W or BacL-CdE/W-CEO, or co-transduced with BacCre and the substrate BV, and were passaged every 2–3 days.

The flow cytometry analysis (Figure 4B) depicted that all substrate vectors gave high %GFP+ cells (≈92–96%) at 1 dpt but BacCre + BacL-CdE/W only led to transient expression. Surprisingly, neither S/MAR nor lamin B2 *ori* supported effective minicircle replication in HEK293 as the %GFP+ cells in the BacCre + BacL-CdE/WS and BacCre + BacL-CdE/W-hO groups also descended precipitously to <1% at 10 dpt. In stark contrast, BacCre + BacL-CdE/W-CEO gave rise to substantially higher and more sustainable %GFP^+^ cells and total FI (lower panel, Figure 4B) that lasted for 30 days. Notably, BacL-CdE/W-CEO alone (without recombination and minicircle formation) only led to transient expression. These data proved that BacCre-mediated recombination and the resultant minicircle harboring *oriP*/EBNA1 prolonged and enhanced the transgene expression in actively dividing cells.

To verify whether the sustained expression was attributable to minicircle retention, total cellular DNA from HEK293 cells was extracted at various time points for qPCR analysis, and the data were normalized to those at 1 dpt. In agreement with the transient expression, BacCre + BacL-CdE/W led to a sharp drop of the relative minicircle copy number to <0.1% at 10 dpt (Figure 4C). With the aid of *oriP*/EBNA1, BacCre + BacL-CdE/W-CEO decelerated the decay, as the relative minicircle copy number remained >20% at 5 dpt and persisted for 30 days. In contrast, the BV genome numbers (Figure 4D) in all groups sharply decreased to <1% at 5 dpt and were barely detectable at 10 dpt, indicating rapid BV genome degradation/dilution in HEK293 cells.

To examine whether sporadic transgene integration occurred, the co-transduced HEK293 cells were subjected to fluorescence in situ hybridization (FISH) analysis at 5 dpt using the probe targeting *d2egfp* (Figure 4E). The absence of fluorescent signals in the mock-transduced cells and the distinct signals associated with the chromosomes in H-d2E [a stable HEK293 clone harboring integrated *d2egfp* (18)] validated the accuracy of FISH. Consequently, the fluorescent signals outside the chromosomes in the BacCre + BacL-CdE/W-CEO group confirmed the episomal form of the recombined minicircles.

### Prolonged growth factor expression and accelerated osteogenesis in hASCs

**Figure 5.**
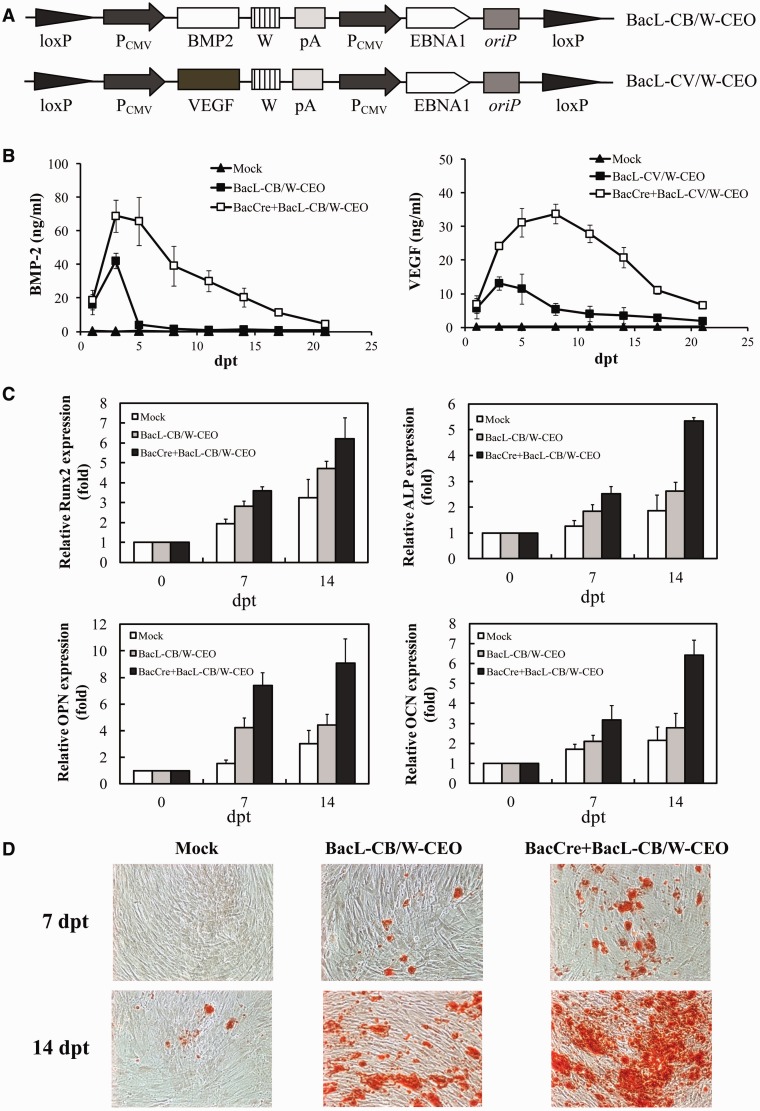
Prolonged growth factor expression and acceleration of osteogenesis. (**A**) Schematic illustration of BacL-CB/W-CEO and BacL-CV/W-CEO. (**B**) Duration of BMP2 and VEGF expression as measured by ELISA. hASCs were mock-transduced, singly transduced with the growth factor-expressing hybrid BV (MOI 200) or co-transduced with BacCre (MOI 100) and the hybrid BV (MOI 200). (**C**) Transcription profiles of osteogenesis markers. The mRNA levels of four osteogenesis marker genes were measured by qRT-PCR at 7 and 14 dpt and normalized against those at 0 dpt. (**D**) Calcium deposition as stained by Alizarin red. Magnification, 100×.

Sustained expression of BMP2 and VEGF is essential to induce the osteogenesis of rASCs and bone regeneration *in vivo* (10). Such prolonged growth factor expression is presumably more critical in hASCs, as hASCs are even more refractory to osteogenic differentiation (31). Therefore, we constructed BacL-CB/W-CEO and BacL-CV/W-CEO that harbored *bmp2* and *vegf*, respectively, using BacL-CdE/W-CEO as the backbone (Figure 5A). hASCs were transduced and cultured in osteoinduction medium without serial passaging because hASCs undergoing differentiation stopped proliferation.

ELISA analysis of the medium (Figure 5B) depicted that BacL-CB/W-CEO alone (MOI 200) gave rise to BMP2 expression that peaked at 3 dpt (≈42 ng/ml) and extinguished at 8 dpt. Similarly, BacL-CV/W-CEO alone conferred VEGF expression that peaked at 3 dpt (≈13 ng/ml) and declined to nearly background levels (<3 ng/ml) after 12 dpt, demonstrating that without recombination, the hybrid BV vector only mediated transient expression, despite the presence of *oriP*/EBNA1. In contrast, co-transduction with BacCre (MOI 100) and BacL-CB/W-CEO (MOI 200) remarkably enhanced the maximum BMP2 level (≈68 ng/ml) at 3 dpt and extended the BMP2 expression to 21 dpt. Likewise, BacCre + BacL-CV/W-CEO elevated the peak VEGF levels (33 ng/ml) and prolonged the expression duration beyond 21 days, attesting that the minicircle formation prolonged and enhanced the growth factor expression.

The qRT-PCR analysis (Figure 5C) further delineated that, when compared with Mock and BacL-CB/W-CEO alone, BacCre + BacL-CB/W-CEO progressively and significantly (*P* < 0.05) upregulated the key osteogenesis marker genes encoding Runx2, alkaline phosphatase, osteopontin and osteocalcin. The Alizarin red staining (Figure 5D), which stains calcium deposition and indicates mineralization, barely detected mineralization in the mock-transduced cells and proved the poor osteogenesis potential of hASCs even in the absence of osteoinduction medium. In contrast, BacCre + BacL-CB/W-CEO provoked denser calcium deposition, hence confirming that BacCre + BacL-CB/W-CEO, and the minicircle formation, accelerated and augmented the osteogenesis of hASCs.

## DISCUSSION

The goal of this study was to develop hybrid BV vectors for prolonged transgene expression in proliferating cells, and non-proliferating stem cells, which may benefit the cell/tissue engineering. Although lentivirus is a common choice for long-term expression, its biased integration into active genes (32) and possible induction of insertional mutagenesis (33) raise safety concerns. Conversely, targeted genome editing can be attained by using zinc finger nuclease (34) or transcription activator-like (TAL) effector nucleases (35), but these nucleases mediate integration at low efficiencies and require antibiotic selection of cells, which may preclude their uses in regenerative medicine because immediate stem cell implantation following genetic modification is desired. In the BV setting, attempts to incorporate adeno-associated virus (AAV) inverted terminal repeats (2,36) or *Sleeping Beauty* transposon (18,19) into BV vectors have been made. For instance, a hybrid BV exploiting the *Sleeping Beauty* transposon system was developed to extend the expression of anti-angiogenic factors for anti-cancer therapy (19). However, these vectors still rely on integration and may not be suitable for stem cell engineering and tissue regeneration. In contrast to these integrating vectors, extrachromosomal maintenance of the transgene is less prone to gene silencing and insertional mutagenesis (24). Therefore, a hybrid BV vector taking advantage of FLP/Frt-mediated recombination was developed to maintain the transgene as a minicircle (37). However, the recombination efficiency is relatively low (40–50%) in rASCs (10).

To augment the recombination and maintain the minicircle, several recombinase systems and *cis*-acting elements were evaluated. We chose ΦC31o, FLPo and Cre because they are commonly used for site-specific integration (23,38), but their efficiencies for generating minicircles have yet to be compared. We uncovered that, in the context of BV genome, ΦC31o was the least efficient (efficiencies ≈40–75%), whereas Cre and FLPo conferred recombination efficiencies up to ≈90–95% (Figure 1D). Considering the integration efficiencies of ΦC31 (<5%), ΦC31o (≈80%), FLP (<5%), FLPo (≈60%) and Cre (≈80%) after antibiotics selection (23), these recombination efficiencies in the absence of selection are strikingly high. Unlike recombinase-mediated gene integration, which is thermodynamically unfavorable (39) and is impeded by the local chromatin structure, BV genome is smaller and is unlikely to deny the access of recombinase to the recognition site on the substrate vector. The Cre-mediated recombination efficiency is also significantly higher than the recombination efficiencies (<40%) mediated by adenovirus-delivered Cre (40,41), presumably owing to at least two reasons. First, recombinases catalyze the excision/recombination in the nucleus; thus, nuclear entry is a prerequisite. BV enters mammalian cells and translocates into nucleus effectively (42), thus permitting the co-entry of the recombinase-expressing BV and substrate BV genomes into the same nuclei for the recombination. Second, circular DNA is more amenable to recombinase-mediated excision/recombination than linear DNA (43). The circular nature of BV genome, in comparison with the linear adenovirus genome, may render BV genome a favorable substrate for the recombinase binding and ensuing recombination.

Furthermore, the BacFLPo- and BacCre-induced recombination was independent of the insert size within 15 kb (Figure 2B), and up to ≈35–50% of the substrate vector underwent recombination (Figure 2C). Such size independency is supported by the report that FLP can efficiently excise a 24–28 kb cassette between Frt sites (44), whereas Cre can excise a megabase-sized DNA fragment flanked by loxP sites (45). Moreover, BacCre and BacFLPo mediated equally efficient recombination/minicircle formation in cell lines/stem cells from different tissues/species (Figure 3A and B), at least partly because Cre and FLPo activities do not need cellular co-factors (39). Importantly, negative influence on cell viability was only appreciable in certain BacFLPo-transduced stem cells, but not in BacCre-transduced cells (Figure 3C), although Cre is known to induce cytotoxicity on continuous expression at high levels (46). The negligible negative effect on BacCre-transduced cells was attributable to the transient Cre expression, which can avoid the cytotoxicity (46), and rendered Cre/loxP a more favorable system in these cells.

To facilitate the maintenance of minicircles in proliferating cells, we incorporated different *cis*-acting elements in the Cre/loxP-based hybrid BV vectors (Figure 4A). However, appending S/MAR or lamin B2 *ori* failed to extend the transgene expression in HEK293 cells (Figure 4B), which was also observed when the constructs were transfected into HEK293 cells in the plasmid form (Supplementary Figure S4), indicating that the phenomenon was not specific for the BV vector. It is documented that inclusion of S/MAR into plasmid facilitates stable episome maintenance (24), but an initial antibiotic selection phase is required (25,47). To avoid sporadic BV genomic integration and potential genotoxicity, no antibiotic selection was implemented in this study, which thus accounted for the failure of S/MAR. Conversely, DNA replication from lamin B2 *ori* requires the formation of pre-replication complex and subsequent binding to the *ori* (48). The failure of lamin B2 *ori* probably resulted from inefficient pre-replication complex formation and binding to the *ori* located on the episomal minicircle.

Among the three elements, only *oriP*/EBNA1 successfully prolonged the transgene expression (Figure 4B) and minicircle maintenance (Figure 4C) in the actively dividing HEK293 cells, which was attributed to the binding of EBNA1 to *oriP* for replication and segregation to daughter cells (49). However, the transgene expression and number of minicircles still diminished and nearly vanished at 30 dpt because without selection episomes harboring *oriP*/EBNA1 are still lost at a rate of ≈2–4% per cell division (50). Critically, after shedding of the payload, the BV genome was quickly degraded in 10 days (Figure 4D), whereas the minicircles existed in the cells in the episomal form without inadvertent integration into the chromosome (Figure 4E), thus warranting its safe use in mammalian cells.

In non-proliferating hASCs, the Cre/loxP-mediated formation of minicircles containing *bmp2*/*vegf* cassettes (Figure 5A) substantially prolonged and enhanced the BMP2/VEGF expression when compared with the expression mediated by the transgene in the BV backbone (Figure 5B). The duration and magnitude of transgene expression increased with the elevated recombination efficiency (Supplementary Figure S5); thus, high recombination efficiency is important. One possible reason for this phenomenon is that the recombination brings *oriP* that has enhancer activities (51) into closer proximity to the CMV promoter and therefore augments the transcription. However, significant enhancement of transgene expression was also observed in the FLPo-based system lacking *oriP*/EBNA1 (unpublished data); thus, the enhancer activity of *oriP* is not the key determinant for the enhanced expression. Conversely, it was shown that minicircles confer stronger and longer transgene expression than their plasmid counterpart (52), which was associated with chromatin-linked transcription blockade (53) but was independent of CpG methylation (52). As BV genome (134 kb) is significantly larger than the minicircle (<10 kb), one may envisage that BV genome, when compared with the smaller minicircle, forms a chromatin structure that less favors persistent transgene expression. However, the exact mechanisms contributing to the differential expression await further investigations. Besides, the hybrid BV harboring *oriP*/EBNA1 alone did not further prolong the gene expression because *oriP*/EBNA1 function hinges on cell division. As EBNA1 itself has oncogenic properties (54), *oriP*/EBNA1 may be removed from the hybrid BV vector in the context of stem cell engineering.

Altogether, by comparing different recombinases and *cis*-acting elements, we developed a binary hybrid BV system featured with Cre/loxP-mediated recombination and *oriP*/EBNA1. Although *oriP*/EBNA1-based replicon can be delivered via herpes simplex virus type 1 (55) or adenovirus vector (56), herpes simplex virus type 1 is immunogenic and toxic to non-neuronal cells, whereas adenovirus mounts strong immune responses. In contrast, the hybrid BV conferred negligible side effects, efficient minicircle formation and maintenance in various cells. These attributes render the hybrid BV an attractive system to deliver exogenous genes into these cells for different applications. For instance, minicircle vector has been used to generate iPS cells (57), but the reprogramming efficiency is limited by the low transfection efficiency for the primary cells. As BV can deliver essential transcription factor genes for the generation of iPS cells (58), it is tempting to use the hybrid BV for highly efficient *in situ* generation of minicircles containing essential transcription factor genes in primary cells to enhance the reprogramming efficiency. Furthermore, AAV and lentivirus vectors are commonly produced by transfection of producer cells, but the procedures are inefficient and costly. As genes necessary for AAV/lentivirus production can be efficiently delivered by BV into producer cells (59,60), the minicircle-associated expression prolongation and enhancement offered by this hybrid BV vector can be used to produce AAV/lentivirus vectors for a longer period with enhanced yield and reduced cost. Finally, ASCs are capable of differentiation into chondrogenic, osteogenic or cardiomyogenic lineages (61), the hybrid BV conferred remarkably high recombination efficiency and persistent growth factor expression, which would facilitate hASCs differentiation toward selected lineages, thereby expanding its applications to cartilage, bone or heart engineering.

One potential drawback of this system is the need for two BV vectors: one expressing Cre while the other harboring the *loxP*-flanking transgene cassette. Developing a one vector system by combining the two cassettes in a single BV may greatly benefit future applications. However, this design requires stringent shutdown of Cre expression during the production of such single BV in insect cells to maintain the genome stability. The CMV-IE promoter driving the Cre expression is weakly active in insect cells (62) and may result in genome instability. Experiments to evaluate different promoters suitable for the construction of such one vector system are ongoing.

## SUPPLEMENTARY DATA

Supplementary Data are available at NAR Online: Supplementary Tables 1–3, Supplementary Figures 1–5 and Supplementary Methods.

## FUNDING

Funding for open access charge: National Tsing Hua University (Toward World-Class University Project [102N2051E1] and NTHU-CGMH Joint Research Program [102N2766E1]) and National Science Council, Taiwan [101-2628-E-007-009-MY3, 101-2325-B-080-001 and 101-2923-E-007-002-MY3].

*Conflict of interest statement.* None declared.

## Supplementary Material

Supplementary Data
