# Supplementary material for: Enhanced and prolonged baculovirus-mediated expression by incorporating recombinase system and in cis elements: a comparative study
Source: Nucleic Acids Res. 2013 May 28;41(14):e139. doi: 10.1093/nar/gkt442 (PMC3737544; doi:10.1093/nar/gkt442)
Supplement: Supplementary Data [file supp_41_14_e139__index.html]

Enhanced and prolonged baculovirus-mediated expression by incorporating recombinase system and in cis elements: a comparative study — Enhanced and prolonged baculovirus-mediated expression by incorporating recombinase system and in cis elements: a comparative study — Supplementary Data 

# Enhanced and prolonged baculovirus-mediated expression by incorporating recombinase system and *in cis* elements: a comparative study

## Supplementary Data

files

**Files in this Data Supplement:**

- Supplementary Data - doc file
