# Supplementary material for: Enhanced and prolonged baculovirus-mediated expression by incorporating recombinase system and in cis elements: a comparative study
Source: Nucleic Acids Res. 2013 May 28;41(14):e139. doi: 10.1093/nar/gkt442 (PMC3737544; doi:10.1093/nar/gkt442)
Supplement: Supplementary Data [file supp_gkt442_nar-00857-met-g-2013-File007.doc]

Supplementary Figures, Tables and Methods

P1

**Minicircle**

**(15 kb)**

P2

**Cre**

**/**

**FLPo**

BacALF

-

15 k

attP / loxP/ Frt

d2

EGFP

p

A

Stuffer

(13 kb)

P_CMV_

attB / loxP/ Frt

A/L/F

d2

EGFP

p

A

Stuffer

DNA

P_CMV_

P1

P2

**pBacALF**

**-**

**CdE**

**(for STD)**

P2

A/L/F

d2

EGFP

p

A

P_CMV_

P1

**Fig. S1.** Primer design strategy for absolute qPCR analysis as shown in Fig. 2. Using BacALF-15k as an example, the co-transduction of BacCre or BacFLPo with BacALF-15k led to the formation of a 15 kb minicircle. Total copy number of *d2egfp* , either on the BV genome or on the minicircle, was measured by qPCR using primer set 1 (P1) specific for *d2egfp*. The minicircle copy number was determined by qPCR using primer pair 2 (P2), which allowed for the generation of PCR products only after minicircles were formed. pBacALF-CdE was constructed and used as a standard (STD) in the qPCR assay. Primer sequences are listed in Table S2.

**Fig. S2.** Primer design strategy for relative qPCR analysis as shown in Fig. 4. After co-transduction with BacCre+BacL-CdE/W-CEO, an 8 kb minicircle was formed. The copy number of total BV genome was measured using primer pair 3 (P3) specific for *gp64*, which is a glycoprotein on the baculoviral envelope. The minicircle copy number was determined using primer pair 4 (P4), for which the forward primer binds to the region between *oriP* and loxP and the reverse primer binds to 5’-end of P_CMV_. The BV and minicircle copy numbers were normalized against those at 1 dpt to yield the relative copy numbers. Primer sequences are listed in Table S2.

P3

P4


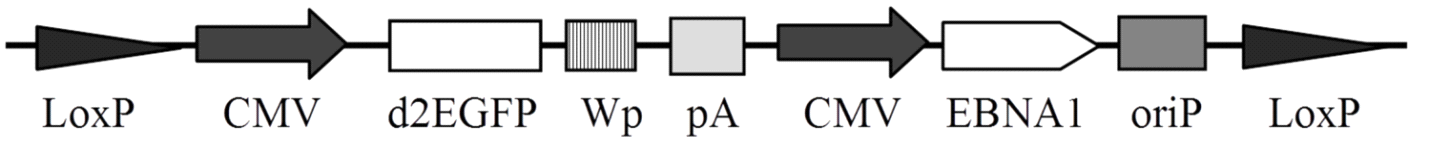


loxP

P_CMV_

d2EGFP

W

pA

**Minicircle (8 kb)**

*oriP*

EBNA1 P_CMV_

P4

**Cre**

g

p64 gene

**BV DNA**

(

BacL

-

CdE

/W

-

CEO)

P_CMV_

P_CMV_

loxP

loxP

*oriP*


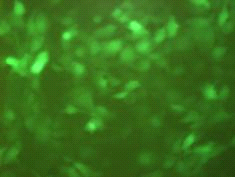

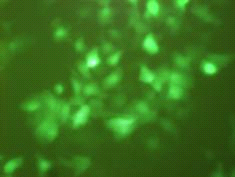

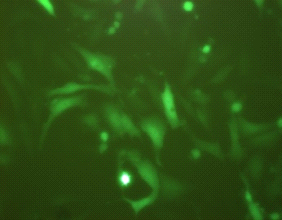

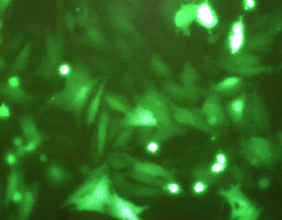

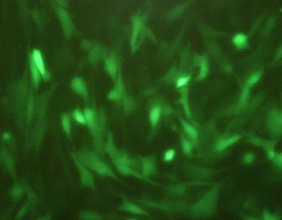

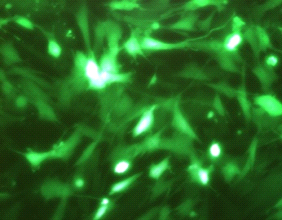

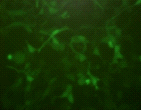

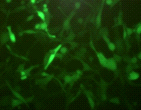

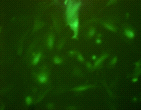

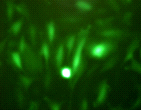

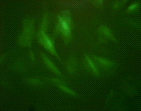

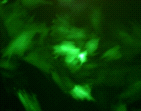


**BacCre+**

**BacALF**

**Huh**

**-**

**7**

**HeLa**

**BHK**

**RD**

**rASCs**

**rBMSCs**

**hASCs**


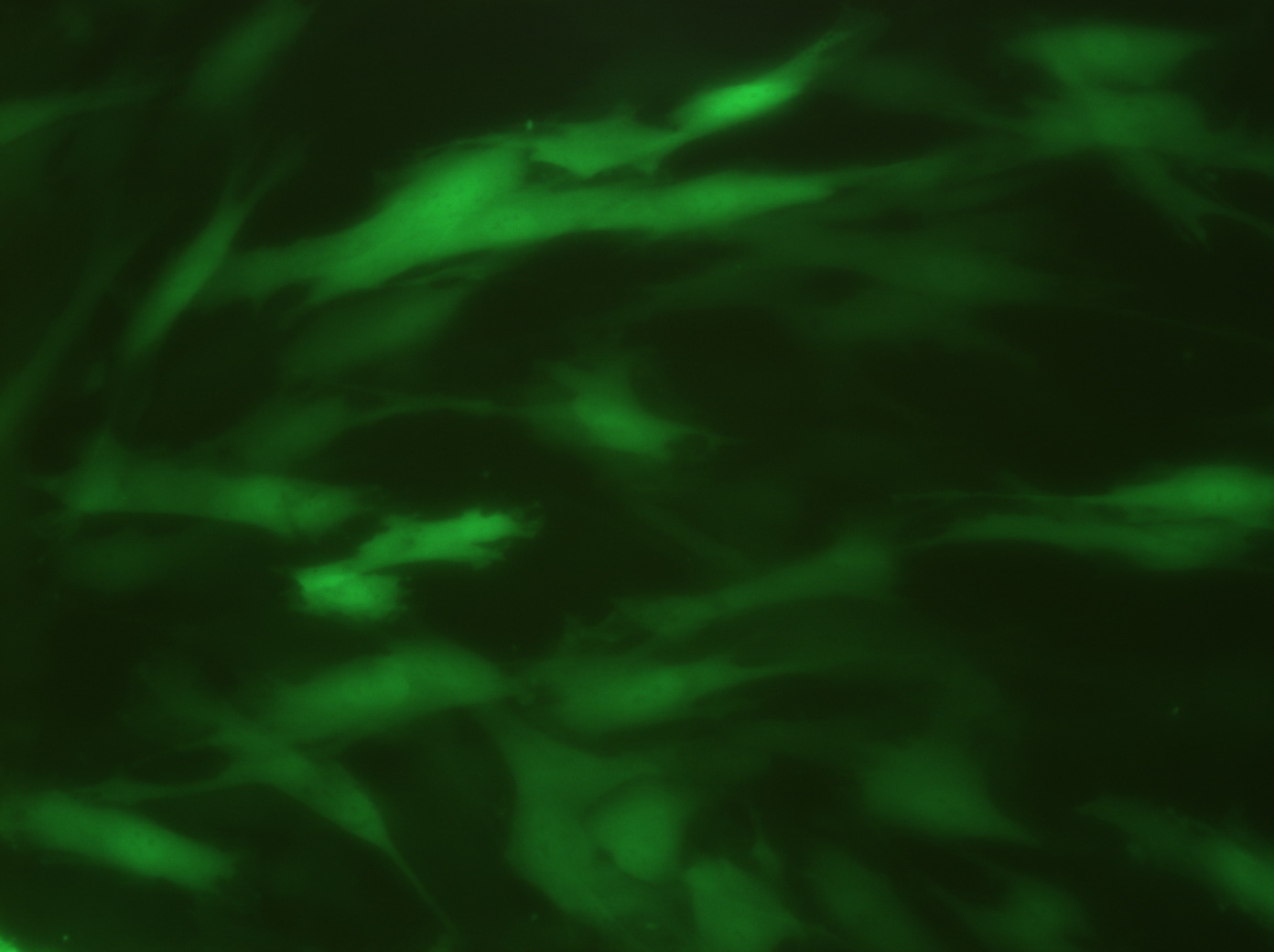

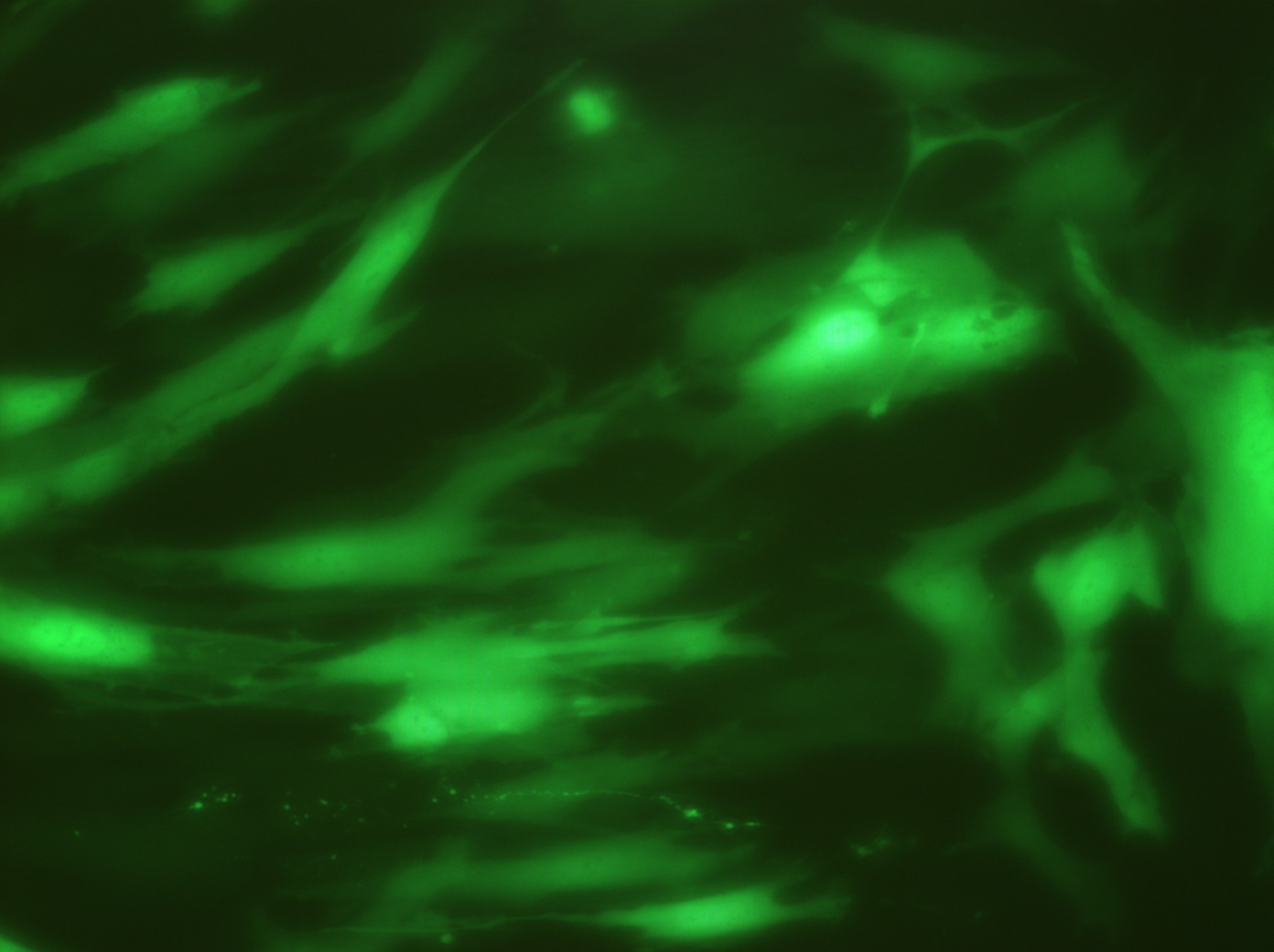


**BacFLPo+**

**BacALF**

**Fig. S3.** Comparison of Cre- and FLPo-mediated recombination in different mammalian cells. Huh-7, HeLa, BHK, RD, rBMSCs, rASCs and hASCs were co-transduced with BacCre (or BacFLPo, MOI 100) and BacALF (MOI 200). At 1 dpt, the d2EGFP expression was monitored by a fluorescence microscope. Magnification, 200X.

10^8^

10^7^

10^6^

10^5^

10^4^

**Fig. S4.** Comparison of transgene expression duration by different *cis*-acting elements via plasmid transfection. HEK293 cells were separately transfected with equal amounts of the plasmids: pBacCdE/W, pBacL-CdE/W-CEO, pBacL-CdE/WS or pBacL-CdE/W-hO. These 4 plasmids are the parental plasmids for the construction of BV vectors shown in Fig. 4A and contained CMV-*d2egfp* cassettes. The latter 3 plasmids additionally harbored *oriP*/EBNA1, S/MAR or human *ori* element. The duration of d2EGFP expression was monitored by flow cytometry. The results of %GFP+ cells (upper panel) and total FI (lower panel) revealed that only pBacL-CdE/W-CEO successfully prolonged transgene expression. No signs of extended transgene expression were found in the pBacL-CdE/WS and pBacL-CdE/W-hO groups, indicating that both S/MAR and human *ori* are not functional (without antibiotic selection). a.u., arbitrary unit.

**(A)**

**(B)**

**Fig. S5.** Enhanced recombination efficiency increased the BMP2 expression duration. (A) Dependency of recombination efficiency on BacCre dose. hASCs were co-transduced with BacCre (MOI 20 or 100) and BacALF (MOI 200) and the %GFP+ cells was analyzed by flow cytometer at 1 dpt. The recombination efficiency was 28% at MOI 20 (of BacCre) and increased to 93% at MOI 100. (B) Effect of recombination efficiency on the duration of BMP2 expression. hASCs were either singly transduced with BacL-CB/W-CEO (MOI 200) or co-transduced with BacCre (MOI 20 or 100) and BacL-CB/W-CEO (MOI 200). The ELISA analysis for BMP2 showed that without recombination (BacL-CB/W-CEO only) the BMP2 expression duration was short. When the recombination efficiency was 28% (BacCre at MOI 20), the BMP2 expression was prolonged to 12 days. When the recombination efficiency was elevated to 93% (BacCre at MOI 100), the BMP2 expression was significantly enhanced and prolonged to 21 days. These data demonstrated that the transgene expression is prolonged and enhanced as the recombination efficiency increases.

**Table S1.** Primer sequences used for construction of recombinant BVs

| **Primer ID** | **Primer sequence (5’🡪3’**, enzyme sites are underlined) |
| --- | --- |
| **Recombinase system** |  |
| ΦC31o _F (EcoRI) | TGAATTCACCATGGATACCTACGCCG |
| ΦC31o _R (NotI) | TGCGGCCGCTCACACTTTCCGCTTTT |
| Cre _F (EcoRI) | AGAATTCACCATGCCCAAGAAGAAGAGG |
| Cre _R (NotI) | TGCGGCCGCTAATCGCCATCTTCCAG |
| FLPo _F (EcoRI) | AGAATTCACCATGGCTCCTAAGAAGAAG |
| FLPo _R (NotI) | TGCGGCCGCTCAGATCCGCCTGTTG |
| d2egfp-pA_F (BamHI) | TTAGGATCCACCATGGTGAGCAAGGG |
| d2egfp-pA _R (BamHI) | TCAGGATCCGGCTCAAGCAGTGATCAGATC |
| CMV_F (StuI) | ATCAGGCCTCCAGATATACGCGTTGACATTG |
| CMV_R (SmaI) | CATCCCGGGTCTAGTTAGCCAGAGAGCTCTG |
| attB-loxP-Frt_F (EcoRI) | CCTAGAATTCGTGCCAGGGCGTGCCCTT |
| attB-loxP-Frt_R (EcoRV) | CTGTGATATCGAAGTTCCTATACTTTCTAGAG |
| ***Cis*-acting element systems** |  |
| WPRE_F (HindIII) | TGACAAGCTTCGATAATCAACCTCTGGATTAC |
| WPRE_R (HindIII) | AGTTAAGCTTCAAAGGGAGATCCGACTCGT |
| loxP_F (SphI/NheI) | CATAACTTCGTATAATGTATGCTATACGAAGTTATG |
| loxP_R (SphI/NheI) | CTAGCATAACTTCGTATAGCATACATTATACGAAGTTATGCATG |
| loxP_F (NotI/PstI) | GGCCGCATAACTTCGTATAATGTATGCTATACGAAGTTATCTGCA |
| loxP_R (NotI/PstI) | GATAACTTCGTATAGCATACATTATACGAAGTTATGC |
| d2egfp-W-pA_F (BamHI) | TTGGATCCACCATGGTGAGCAAGGG |
| d2egfp-W-pA_R (BamHI) | TAGGATCCGGCTCAAGCAGTGATCAGATC |
| S/MAR_F (AvrII) | GCCTAGGAAATAAACTTATAAATTGTGAG |
| S/MAR_R (AvrII) | GCCTAGGAATTCTATCAAGATATTTAAAG |
| W-S/MAR_F (NotI) | TCGCGGCCGCCGATAATCAACCTCTGGATTAC |
| W-S/MAR_R (NotI) | GTGCGGCCGCAATTCTATCAAGATATTTAAAG |
| dE-W-S/MAR-pA_F (BamHI) | TTGGATCCACCATGGTGAGCAAGGG |
| dE-W-S/MAR-pA_R (StuI) | TAAGGCCTGGCTCAAGCAGTGATCAGATC |
| Lamin B2 ori_F (EcoRI) | AAGAATTCAGATCTGAGGGACTCGTCAGTC |
| Lamin B2 ori_R (EcoRI) | CCGAATTCTCGCGACCCTTGCCC |

**Table S2. Primer sequences used in absolute and relative qPCR**

| **Primer ID** | **Primer sequence (5’🡪3’)** |
| --- | --- |
| **Absolute qPCR** |  |
| *d2egfp* (P1)_F | TATATCATGGCCGACAAGCA |
| *d2egfp* (P1)_R | TGTTCTGCTGGTAGTGGTCG |
| Minicircle (P2)_F | GCGTGTACGGTGGGAGGTCTA |
| Minicircle (P2)_R | TCCTCGCCCTTGCTCACCA |
| **Relative qPCR** |  |
| *gp64* (P3)_F | ATGTGGGCAAAGAGGATTTG |
| *gp64* (P3)_R | GAAAACAGTCGTCGCTGTCA |
| Minicircle (P4)_F | GACGAGCTCACTAGTCGCG |
| Minicircle (P4)_R | CTAGTCAATAATCAATGTCAACGC |
| *gapdh*_F | GTCAGTGGTGGACCTGACCT |
| *gapdh*_R | ACCTGGTGCTCAGTGTAGCC |

**Table S3. Primer sequences used in qRT-PCR**

| **Primer ID** | **Primer sequence (5’🡪3’)** |
| --- | --- |
| *gapdh*_F | GTCTCCTCTGACTTCAACAGCG |
| *gapdh*_R | ACCACCCTGTTGCTGTAGCCAA |
| Runx2_F | TGAGAGTAGGTGTCCCGCCT |
| Runx2_R | TGTGGATTAAAAGGACTTGGTGC |
| ALP_F | CCGTGGCAACTCTATCTTTGG |
| ALP_R | GATGGCAGTGAAGGGCTTCTT |
| OPN_F | CTAGGCATCACCTGTGCCATACC |
| OPN_R | CAGTGACCAGTTCATCAGATTCATC |
| OCN_F | GCAGGTGCGAAGCCCA |
| OCN_R | TCCTGCTTGGACACAAAGGC |

Supplementary Materials and Methods

Cell Culture

Mammalian cell lines (HeLa, HEK293, Huh-7, BHK, and RD) were cultured using Dulbecco’s modified eagle’s medium (DMEM, high glucose; Sigma, St Louis, MO) containing 10% fetal bovine serum (FBS; HyClone, Rockford, IL) in T-75 flasks at 37°C. Rabbit ASCs (rASCs) and BMSCs (rBMSCs) were isolated and cultured as described in ref. 8 and 10, respectively. Cells of passage 4 to 6 were used for experiments. Human ASCs (hASCs) were maintained using αMEM medium (Sigma) containing 10% FBS and 4 ng/ml basic fibroblast growth factor (bFGF; PeproTech, Rocky Hill, NJ) in T-75 flasks at 37°C.

Calculation of minicircle formation efficiency

The minicircle formation efficiency was defined as the percentage of the minicircles excised from the substrate BV genome. The index was calculated by dividing the absolute copy number of minicircles to that of *d2egfp* (representing the total copy number of substrate BV entering the cells) for each group.

Real-time quantitative reverse transcription PCR (qRT-PCR)

qRT-PCR was performed to quantify the transcription levels of osteogenic marker genes (Runx2, ALP, OPN and OCN). Total cellular RNA was extracted from hASCs with the NucleoSpin® RNA II Kit (Macherey-Nagel, Duren, Germany), and 1 µg of the RNA was reverse transcribed to cDNA with the Omniscript RT Kit (Qiagen, Hilden, Germany). Five microliter of the diluted cDNA (500x) was used for qPCR reactions, using primer sets specific for the osteogenic marker genes (Table S3). All data were normalized against those at 1 dpt.

Sequences of the tandem recombination sites

1 TCTCGAGCCT CCCCAACTGG GGTAACCTTT GAGTTCTCTC AGTTGGGGGC

XhoI **attP**

51 GAGCTTAAAC ATAACTTCGT ATAATGTATG CTATACGAAG TTATCCGTGA

**loxP**

101 CAGTGAAGTT CCTATTCTCT AGAAAGTATA GGAACTTCCC TGGCGGATCC

**Frt** BamHI

151 CGGAGGCCTA CGAGCTGTGC CAGGGCGTGC CCTTGGGCTC CCCGGGCGCG

StuI **attB**

201 ACTTGGTCGG CATAACTTCG TATAATGTAT GCTATACGAA GTTATGTTAA

**loxP**

251 TGCGACCGAA GTTCCTATTC TCTAGAAAGT ATAGGAACTT CATAAAGCTT

**Frt** HindIII

301 A


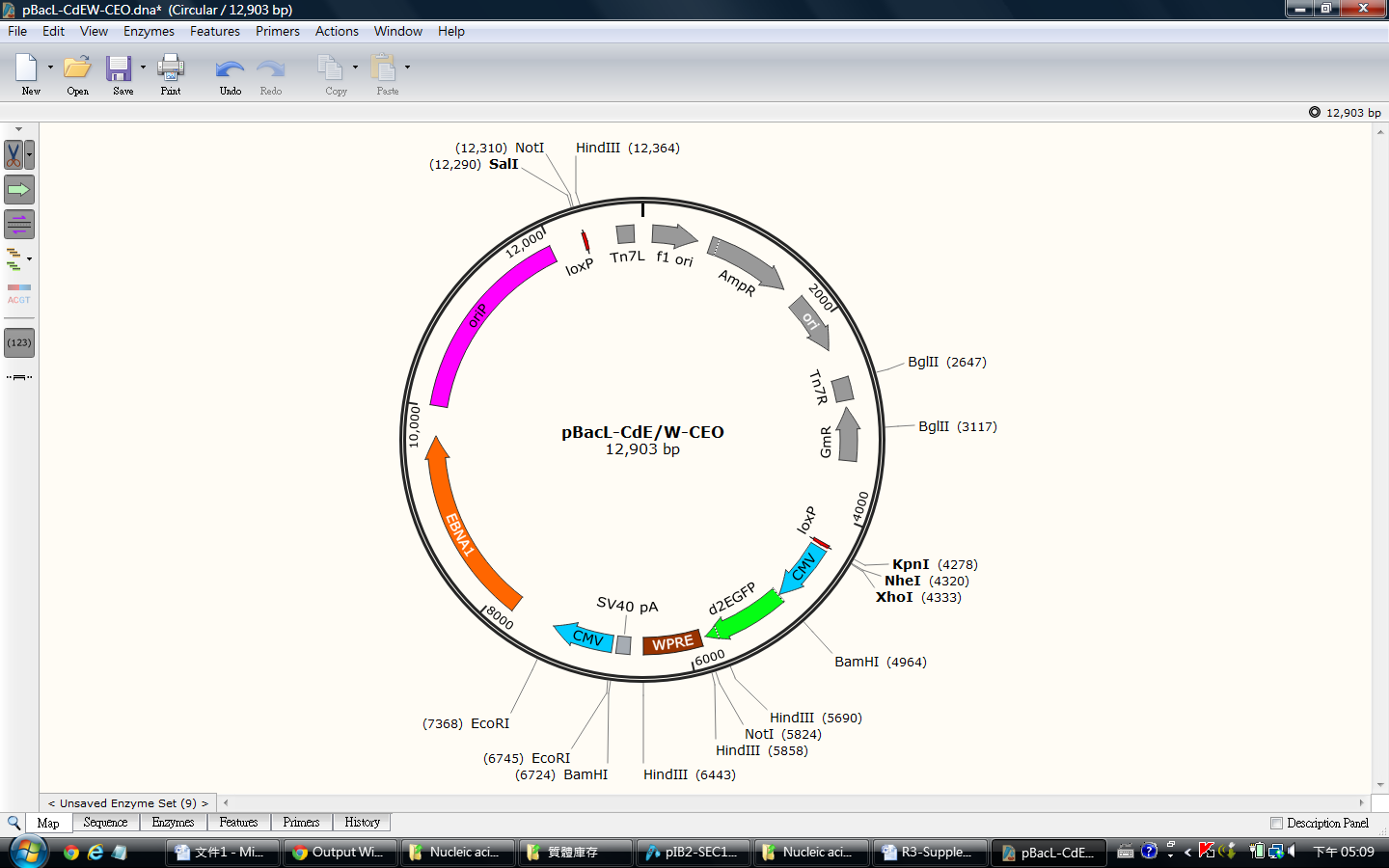
Map and Sequences of pBacL-CdE/W-CEO

TTCTCTGTCACAGAATGAAAATTTTTCTGTCATCTCTTCGTTATTAATGTTTGTAATTGACTGAATATCAACGCTTATTTGCAGCCTGAATGGCGAATGGGACGCGCCCTGTAGCGGCGCATTAAGCGCGGCGGGTGTGGTGGTTACGCGCAGCGTGACCGCTACACTTGCCAGCGCCCTAGCGCCCGCTCCTTTCGCTTTCTTCCCTTCCTTTCTCGCCACGTTCGCCGGCTTTCCCCGTCAAGCTCTAAATCGGGGGCTCCCTTTAGGGTTCCGATTTAGTGCTTTACGGCACCTCGACCCCAAAAAACTTGATTAGGGTGATGGTTCACGTAGTGGGCCATCGCCCTGATAGACGGTTTTTCGCCCTTTGACGTTGGAGTCCACGTTCTTTAATAGTGGACTCTTGTTCCAAACTGGAACAACACTCAACCCTATCTCGGTCTATTCTTTTGATTTATAAGGGATTTTGCCGATTTCGGCCTATTGGTTAAAAAATGAGCTGATTTAACAAAAATTTAACGCGAATTTTAACAAAATATTAACGTTTACAATTTCAGGTGGCACTTTTCGGGGAAATGTGCGCGGAACCCCTATTTGTTTATTTTTCTAAATACATTCAAATATGTATCCGCTCATGAGACAATAACCCTGATAAATGCTTCAATAATATTGAAAAAGGAAGAGTATGAGTATTCAACATTTCCGTGTCGCCCTTATTCCCTTTTTTGCGGCATTTTGCCTTCCTGTTTTTGCTCACCCAGAAACGCTGGTGAAAGTAAAAGATGCTGAAGATCAGTTGGGTGCACGAGTGGGTTACATCGAACTGGATCTCAACAGCGGTAAGATCCTTGAGAGTTTTCGCCCCGAAGAACGTTTTCCAATGATGAGCACTTTTAAAGTTCTGCTATGTGGCGCGGTATTATCCCGTATTGACGCCGGGCAAGAGCAACTCGGTCGCCGCATACACTATTCTCAGAATGACTTGGTTGAGTACTCACCAGTCACAGAAAAGCATCTTACGGATGGCATGACAGTAAGAGAATTATGCAGTGCTGCCATAACCATGAGTGATAACACTGCGGCCAACTTACTTCTGACAACGATCGGAGGACCGAAGGAGCTAACCGCTTTTTTGCACAACATGGGGGATCATGTAACTCGCCTTGATCGTTGGGAACCGGAGCTGAATGAAGCCATACCAAACGACGAGCGTGACACCACGATGCCTGTAGCAATGGCAACAACGTTGCGCAAACTATTAACTGGCGAACTACTTACTCTAGCTTCCCGGCAACAATTAATAGACTGGATGGAGGCGGATAAAGTTGCAGGACCACTTCTGCGCTCGGCCCTTCCGGCTGGCTGGTTTATTGCTGATAAATCTGGAGCCGGTGAGCGTGGGTCTCGCGGTATCATTGCAGCACTGGGGCCAGATGGTAAGCCCTCCCGTATCGTAGTTATCTACACGACGGGGAGTCAGGCAACTATGGATGAACGAAATAGACAGATCGCTGAGATAGGTGCCTCACTGATTAAGCATTGGTAACTGTCAGACCAAGTTTACTCATATATACTTTAGATTGATTTAAAACTTCATTTTTAATTTAAAAGGATCTAGGTGAAGATCCTTTTTGATAATCTCATGACCAAAATCCCTTAACGTGAGTTTTCGTTCCACTGAGCGTCAGACCCCGTAGAAAAGATCAAAGGATCTTCTTGAGATCCTTTTTTTCTGCGCGTAATCTGCTGCTTGCAAACAAAAAAACCACCGCTACCAGCGGTGGTTTGTTTGCCGGATCAAGAGCTACCAACTCTTTTTCCGAAGGTAACTGGCTTCAGCAGAGCGCAGATACCAAATACTGTCCTTCTAGTGTAGCCGTAGTTAGGCCACCACTTCAAGAACTCTGTAGCACCGCCTACATACCTCGCTCTGCTAATCCTGTTACCAGTGGCTGCTGCCAGTGGCGATAAGTCGTGTCTTACCGGGTTGGACTCAAGACGATAGTTACCGGATAAGGCGCAGCGGTCGGGCTGAACGGGGGGTTCGTGCACACAGCCCAGCTTGGAGCGAACGACCTACACCGAACTGAGATACCTACAGCGTGAGCATTGAGAAAGCGCCACGCTTCCCGAAGGGAGAAAGGCGGACAGGTATCCGGTAAGCGGCAGGGTCGGAACAGGAGAGCGCACGAGGGAGCTTCCAGGGGGAAACGCCTGGTATCTTTATAGTCCTGTCGGGTTTCGCCACCTCTGACTTGAGCGTCGATTTTTGTGATGCTCGTCAGGGGGGCGGAGCCTATGGAAAAACGCCAGCAACGCGGCCTTTTTACGGTTCCTGGCCTTTTGCTGGCCTTTTGCTCACATGTTCTTTCCTGCGTTATCCCCTGATTCTGTGGATAACCGTATTACCGCCTTTGAGTGAGCTGATACCGCTCGCCGCAGCCGAACGACCGAGCGCAGCGAGTCAGTGAGCGAGGAAGCGGAAGAGCGCCTGATGCGGTATTTTCTCCTTACGCATCTGTGCGGTATTTCACACCGCAGACCAGCCGCGTAACCTGGCAAAATCGGTTACGGTTGAGTAATAAATGGATGCCCTGCGTAAGCGGGTGTGGGCGGACAATAAAGTCTTAAACTGAACAAAATAGATCTAAACTATGACAATAAAGTCTTAAACTAGACAGAATAGTTGTAAACTGAAATCAGTCCAGTTATGCTGTGAAAAAGCATACTGGACTTTTGTTATGGCTAAAGCAAACTCTTCATTTTCTGAAGTGCAAATTGCCCGTCGTATTAAAGAGGGGCGTGGCCAAGGGCATGGTAAAGACTATATTCGCGGCGTTGTGACAATTTACCGAACAACTCCGCGGCCGGGAAGCCGATCTCGGCTTGAACGAATTGTTAGGTGGCGGTACTTGGGTCGATATCAAAGTGCATCACTTCTTCCCGTATGCCCAACTTTGTATAGAGAGCCACTGCGGGATCGTCACCGTAATCTGCTTGCACGTAGATCACATAAGCACCAAGCGCGTTGGCCTCATGCTTGAGGAGATTGATGAGCGCGGTGGCAATGCCCTGCCTCCGGTGCTCGCCGGAGACTGCGAGATCATAGATATAGATCTCACTACGCGGCTGCTCAAACCTGGGCAGAACGTAAGCCGCGAGAGCGCCAACAACCGCTTCTTGGTCGAAGGCAGCAAGCGCGATGAATGTCTTACTACGGAGCAAGTTCCCGAGGTAATCGGAGTCCGGCTGATGTTGGGAGTAGGTGGCTACGTCTCCGAACTCACGACCGAAAAGATCAAGAGCAGCCCGCATGGATTTGACTTGGTCAGGGCCGAGCCTACATGTGCGAATGATGCCCATACTTGAGCCACCTAACTTTGTTTTAGGGCGACTGCCCTGCTGCGTAACATCGTTGCTGCTGCGTAACATCGTTGCTGCTCCATAACATCAAACATCGACCCACGGCGTAACGCGCTTGCTGCTTGGATGCCCGAGGCATAGACTGTACAAAAAAACAGTCATAACAAGCCATGAAAACCGCCACTGCGCCGTTACCACCGCTGCGTTCGGTCAAGGTTCTGGACCAGTTGCGTGAGCGCATACGCTACTTGCATTACAGTTTACGAACCGAACAGGCTTATGTCAACTGGGTTCGTGCCTTCATCCGTTTCCACGGTGTGCGTCACCCGGCAACCTTGGGCAGCAGCGAAGTCGAGGCATTTCTGTCCTGGCTGGCGAACGAGCGCAAGGTTTCGGTCTCCACGCATCGTCAGGCATTGGCGGCCTTGCTGTTCTTCTACGGCAAGGTGCTGTGCACGGATCTGCCCTGGCTTCAGGAGATCGGTAGACCTCGGCCGTCGCGGCGCTTGCCGGTGGTGCTGACCCCGGATGAAGTGGTTCGCATCCTCGGTTTTCTGGAAGGCGAGCATCGTTTGTTCGCCCAGGACTCTAGCTATAGTTCTAGTGGTTGGCCTACGTACCCGTAGTGGCTATGGCAGGGCTTGCCGCCCCGACGTTGGCTGCGAGCCCTGGGCCTTCACCCGAACTTGGGGGTTGGGGTGGGGAAAAGGAAGAAACGCGGGCGTATTGGTCCCAATGGGGTCTCGGTGGGGTATCGACAGAGTGCCAGCCCTGGGACCGAACCCCGCGTTTATGAACAAACGACCCAACACCCGTGCGTTTTATTCTGTCTTTTTATTGCCGTCATAGCGCGGGTTCCTTCCGGTATTGTCTCCTTCCGTGTTTCAGTTAGCCTCCCCCATCTCCCGGTACCGCATGCATAACTTCGTATAATGTATGCTATACGAAGTTATGCTAGCACCATGGCTCGAGATCCCGGGACATTGATTATTGACTAGTTATTAATAGTAATCAATTACGGGGTCATTAGTTCATAGCCCATATATGGAGTTCCGCGTTACATAACTTACGGTAAATGGCCCGCCTGGCTGACCGCCCAACGACCCCCGCCCATTGACGTCAATAATGACGTATGTTCCCATAGTAACGCCAATAGGGACTTTCCATTGACGTCAATGGGTGGAGTATTTACGGTAAACTGCCCACTTGGCAGTACATCAAGTGTATCATATGCCAAGTACGCCCCCTATTGACGTCAATGACGGTAAATGGCCCGCCTGGCATTATGCCCAGTACATGACCTTATGGGACTTTCCTACTTGGCAGTACATCTACGTATTAGTCATCGCTATTACCATGGTGATGCGGTTTTGGCAGTACATCAATGGGCGTGGATAGCGGTTTGACTCACGGGGATTTCCAAGTCTCCACCCCATTGACGTCAATGGGAGTTTGTTTTGGCACCAAAATCAACGGGACTTTCCAAAATGTCGTAACAACTCCGCCCCATTGACGCAAATGGGCGGTAGGCGTGTACGGTGGGAGGTCTATATAAGCAGAGCTCTCTGGCTAACTAGAGAACCCACTGCTTACTGGCGGATCCACCATGGTGAGCAAGGGCGAGGAGCTGTTCACCGGGGTGGTGCCCATCCTGGTCGAGCTGGACGGCGACGTAAACGGCCACAAGTTCAGCGTGTCCGGCGAGGGCGAGGGCGATGCCACCTACGGCAAGCTGACCCTGAAGTTCATCTGCACCACCGGCAAGCTGCCCGTGCCCTGGCCCACCCTCGTGACCACCCTGACCTACGGCGTGCAGTGCTTCAGCCGCTACCCCGACCACATGAAGCAGCACGACTTCTTCAAGTCCGCCATGCCCGAAGGCTACGTCCAGGAGCGCACCATCTTCTTCAAGGACGACGGCAACTACAAGACCCGCGCCGAGGTGAAGTTCGAGGGCGACACCCTGGTGAACCGCATCGAGCTGAAGGGCATCGACTTCAAGGAGGACGGCAACATCCTGGGGCACAAGCTGGAGTACAACTACAACAGCCACAACGTCTATATCATGGCCGACAAGCAGAAGAACGGCATCAAGGTGAACTTCAAGATCCGCCACAACATCGAGGACGGCAGCGTGCAGCTCGCCGACCACTACCAGCAGAACACCCCCATCGGCGACGGCCCCGTGCTGCTGCCCGACAACCACTACCTGAGCACCCAGTCCGCCCTGAGCAAAGACCCCAACGAGAAGCGCGATCACATGGTCCTGCTGGAGTTCGTGACCGCCGCCGGGATCACTCTCGGCATGGACGAGCTGTACAAGAAGCTTAGCCATGGCTTCCCGCCGGAGGTGGAGGAGCAGGATGATGGCACGCTGCCCATGTCTTGTGCCCAGGAGAGCGGGATGGACCGTCACCCTGCAGCCTGTGCTTCTGCTAGGATCAATGTGTAGATGCGCGGCCGCGTTTCGAATCTAGAGTGCAGTCTCGACAAGCTTCGATAATCAACCTCTGGATTACAAAATTTGTGAAAGATTGACTGGTATTCTTAACTATGTTGCTCCTTTTACGCTATGTGGATACGCTGCTTTAATGCCTTTGTATCATGCTATTGCTTCCCGTATGGCTTTCATTTTCTCCTCCTTGTATAAATCCTGGTTGCTGTCTCTTTATGAGGAGTTGTGGCCCGTTGTCAGGCAACGTGGCGTGGTGTGCACTGTGTTTGCTGACGCAACCCCCACTGGTTGGGGCATTGCCACCACCTGTCAGCTCCTTTCCGGGACTTTCGCTTTCCCCCTCCCTATTGCCACGGCGGAACTCATCGCCGCCTGCCTTGCCCGCTGCTGGACAGGGGCTCGGCTGTTGGGCACTGACAATTCCGTGGTGTTGTCGGGGAAGCTGACGTCCTTTCCATGGCTGCTCGCCTGTGTTGCCACCTGGATTCTGCGCGGGACGTCCTTCTGCTACGTCCCTTCGGCCCTCAATCCAGCGGACCTTCCTTCCCGCGGCCTGCTGCCGGCTCTGCGGCCTCTTCCGCGTCTTCGCCTTCGCCCTCAGACGAGTCGGATCTCCCTTTGAAGCTTGTCGAGAAGTACTAGAGGATCATAATCAGCCATACCACATTTGTAGAGGTTTTACTTGCTTTAAAAAACCTCCCACACCTCCCCCTGAACCTGAAACATAAAATGAATGCAATTGTTGTTGTTAACTTGTTTATTGCAGCTTATAATGGTTACAAATAAAGCAATAGCATCACAAATTTCACAAATAAAGCATTTTTTTCACTGCATTCTAGTTGTGGTTTGTCCAAACTCATCAATGTATCTTATCATGTCTGGATCTGATCACTGCTTGAGCCGGATCCGGTCCGAAGCGCGCGGAATTCACATTGATTATTGACTAGTTATTAATAGTAATCAATTACGGGGTCATTAGTTCATAGCCCATATATGGAGTTCCGCGTTACATAACTTACGGTAAATGGCCCGCCTGGCTGACCGCCCAACGACCCCCGCCCATTGACGTCAATAATGACGTATGTTCCCATAGTAACGCCAATAGGGACTTTCCATTGACGTCAATGGGTGGAGTATTTACGGTAAACTGCCCACTTGGCAGTACATCAAGTGTATCATATGCCAAGTACGCCCCCTATTGACGTCAATGACGGTAAATGGCCCGCCTGGCATTATGCCCAGTACATGACCTTATGGGACTTTCCTACTTGGCAGTACATCTACGTATTAGTCATCGCTATTACCATGGTGATGCGGTTTTGGCAGTACATCAATGGGCGTGGATAGCGGTTTGACTCACGGGGATTTCCAAGTCTCCACCCCATTGACGTCAATGGGAGTTTGTTTTGGCACCAAAATCAACGGGACTTTCCAAAATGTCGTAACAACTCCGCCCCATTGACGCAAATGGGCGGTAGGCGTGTACGGTGGGAGGTCTATATAAGCAGAGCTCTCTGGCTAACTAGAGAACCCACTGCTTACTGGCGAATTCTCATGTTTGACAGCTTATCATCGATAAGCTGATCCTCACAGGCCGCACCCAGCTTTTCTTCCGTTGCCCCAGTAGCATCTCTGTCTGGTGACCTTGAAGAGGAAGAGGAGGGGTCCCGAGAATCCCCATCCCTACCGTCCAGCAAAAAGGGGGACGAGGAATTTGAGGCCTGGCTTGAGGCTCAGGACGCAAATCTTGAGGATGTTCAGCGGGAGTTTTCCGGGCTGCGAGTAATTGGTGATGAGGACGAGGATGGTTCGGAGGATGGGGAATTTTCAGACCTGGATCTGTCTGACAGCGACCATGAAGGGGATGAGGGTGGGGGGGCTGTTGGAGGGGGCAGGAGTCTGCACTCCCTGTATTCACTGAGCGTCGTCTAATAAAGATGTCTATTGATCTCTTTTAGTGTGAATCATGTCTGACGAGGGGCCAGGTACAGGACCTGGAAATGGCCTAGGAGAGAAGGGAGACACATCTGGACCAGAAGGCTCCGGCGGCAGTGGACCTCAAAGAAGAGGGGGTGATAACCATGGACGAGGACGGGGAAGAGGACGAGGACGAGGAGGCGGAAGACCAGGAGCCCCGGGCGGCTCAGGATCAGGGCCAAGACATAGAGATGGTGTCCGGAGACCCCAAAAACGTCCAAGTTGCATTGGCTGCAAAGGGACCCACGGTGGAACAGGAGCAGGAGCAGGAGCGGGAGGGGCAGGAGCAGGAGGGGCAGGAGCAGGAGGAGGGGCAGGAGCAGGAGGAGGGGCAGGAGGGGCAGGAGGGGCAGGAGGGGCAGGAGCAGGAGGAGGGGCAGGAGCAGGAGGAGGGGCAGGAGGGGCAGGAGGGGCAGGAGCAGGAGGAGGGGCAGGAGCAGGAGGAGGGGCAGGAGGGGCAGGAGCAGGAGGAGGGGCAGGAGGGGCAGGAGGGGCAGGAGCAGGAGGAGGGGCAGGAGCAGGAGGAGGGGCAGGAGGGGCAGGAGCAGGAGGAGGGGCAGGAGGGGCAGGAGGGGCAGGAGCAGGAGGAGGGGCAGGAGCAGGAGGGGCAGGAGGGGCAGGAGGGGCAGGAGCAGGAGGGGCAGGAGCAGGAGGAGGGGCAGGAGGGGCAGGAGGGGCAGGAGCAGGAGGGGCAGGAGCAGGAGGGGCAGGAGCAGGAGGGGCAGGAGCAGGAGGGGCAGGAGGGGCAGGAGCAGGAGGGGCAGGAGGGGCAGGAGCAGGAGGGGCAGGAGGGGCAGGAGCAGGAGGAGGGGCAGGAGGGGCAGGAGCAGGAGGAGGGGCAGGAGGGGCAGGAGCAGGAGGGGCAGGAGGGGCAGGAGCAGGAGGGGCAGGAGGGGCAGGAGCAGGAGGGGCAGGAGGGGCAGGAGCAGGAGGAGGGGCAGGAGCAGGAGGGGCAGGAGCAGGAGGTGGAGGCCGGGGTCGAGGAGGCAGTGGAGGCCGGGGTCGAGGAGGTAGTGGAGGCCGGGGTCGAGGAGGTAGTGGAGGCCGCCGGGGTAGAGGACGTGAAAGAGCCAGGGGGGGAAGTCGTGAAAGAGCCAGGGGGAGAGGTCGTGGACGTGGAGAAAAGAGGCCCAGGAGTCCCAGTAGTCAGTCATCATCATCCGGGTCTCCACCGCGCAGGCCCCCTCCAGGTAGAAGGCCATTTTTCCACCCTGTAGGGGAAGCCGATTATTTTGAATACCACCAAGAAGGTGGCCCAGATGGTGAGCCTGACGTGCCCCCGGGAGCGATAGAGCAGGGCCCCGCAGATGACCCAGGAGAAGGCCCAAGCACTGGACCCCGGGGTCAGGGTGATGGAGGCAGGCGCAAAAAAGGAGGGTGGTTTGGAAAGCATCGTGGTCAAGGAGGTTCCAACCCGAAATTTGAGAACATTGCAGAAGGTTTAAGAGCTCTCCTGGCTAGGAGTCACGTAGAAAGGACTACCGACGAAGGAACTTGGGTCGCCGGTGTGTTCGTATATGGAGGTAGTAAGACCTCCCTTTACAACCTAAGGCGAGGAACTGCCCTTGCTATTCCACAATGTCGTCTTACACCATTGAGTCGTCTCCCCTTTGGAATGGCCCCTGGACCCGGCCCACAACCTGGCCCGCTAAGGGAGTCCATTGTCTGTTATTTCATGGTCTTTTTACAAACTCATATATTTGCTGAGGTTTTGAAGGATGCGATTAAGGACCTTGTTATGACAAAGCCCGCTCCTACCTGCAATATCAGGGTGACTGTGTGCAGCTTTGACGATGGAGTAGATTTGCCTCCCTGGTTTCCACCTATGGTGGAAGGGGCTGCCGCGGAGGGTGATGACGGAGATGACGGAGATGAAGGAGGTGATGGAGATGAGGGTGAGGAAGGGCAGGAGTGATGTAACTTGTTAGGAGACGCCCTCAATCGTATTAAAAGCCGTGTATTCCCCCGCACTAAAGAATAAATCCCCAGTAGACATCATGCGTGCTGTTGGTGTATTTCTGGCCATCTGTCTTGTCACCATTTTCGTCCTCCCAACATGGGGCAATTGGGCATACCCATGTTGTCACGTCACTCAGCTCCGCGCTCAACACCTTCTCGCGTTGGAAAACATTAGCGACATTTACCTGGTGAGCAATCAGACATGCGACGGCTTTAGCCTGGCCTCCTTAAATTCACCTAAGAATGGGAGCAACCAGCAGGAAAAGGACAAGCAGCGAAAATTCACGCCCCCTTGGGAGGTGGCGGCATATGCAAAGGATAGCACTCCCACTCTACTACTGGGTATCATATGCTGACTGTATATGCATGAGGATAGCATATGCTACCCGGATACAGATTAGATAGCATATACTACCCAGATATAGATTAGGATAGCATATGCTACCCAGATATAGATTAGGATAGCCTATGCTACCCAGATATAAATTAGGATAGCATATACTACCCAGATATAGATTAGGATAGCATATGCTACCCAGATATAGATTAGGATAGCCTATGCTACCCAGATATAGATTAGGATAGCATATGCTACCCAGATATAGATTAGGATAGCATATGCTATCCAGATATTTGGGTAGTATATGCTACCCAGATATAAATTAGGATAGCATATACTACCCTAATCTCTATTAGGATAGCATATGCTACCCGGATACAGATTAGGATAGCATATACTACCCAGATATAGATTAGGATAGCATATGCTACCCAGATATAGATTAGGATAGCCTATGCTACCCAGATATAAATTAGGATAGCATATACTACCCAGATATAGATTAGGATAGCATATGCTACCCAGATATAGATTAGGATAGCCTATGCTACCCAGATATAGATTAGGATAGCATATGCTATCCAGATATTTGGGTAGTATATGCTACCCATGGCAACATTAGCCCACCGTGCTCTCAGCGACCTCGTGAATATGAGGACCAACAACCCTGTGCTTGGCGCTCAGGCGCAAGTGTGTGTAATTTGTCCTCCAGATCGCAGCAATCGCGCCCCTATCTTGGCCCGCCCACCTACTTATGCAGGTATTCCCCGGGGTGCCATTAGTGGTTTTGTGGGCAAGTGGTTTGACCGCAGTGGTTAGCGGGGTTACAATCAGCCAAGTTATTACACCCTTATTTTACAGTCCAAAACCGCAGGGCGGCGTGTGGGGGCTGACGCGTGCCCCCACTCCACAATTTCAAAAAAAAGAGTGGCCACTTGTCTTTGTTTATGGGCCCCATTGGCGTGGAGCCCCGTTTAATTTTCGGGGGTGTTAGAGACAACCAGTGGAGTCCGCTGCTGTCGGCGTCCACTCTCTTTCCCCTTGTTACAAATAGAGTGTAACAACATGGTTCACCTGTCTTGGTCCCTGCCTGGGACACATCTTAATAACCCCAGTATCATATTGCACTAGGATTATGTGTTGCCCATAGCCATAAATTCGTGTGAGATGGACATCCAGTCTTTACGGCTTGTCCCCACCCCATGGATTTCTATTGTTAAAGATATTCAGAATGTTTCATTCCTACACTAGTATTTATTGCCCAAGGGGTTTGTGAGGGTTATATTGGTGTCATAGCACAATGCCACCACTGAACCCCCCGTCCAAATTTTATTCTGGGGGCGTCACCTGAAACCTTGTTTTCGAGCACCTCACATACACCTTACTGTTCACAACTCAGCAGTTATTCTATTAGCTAAACGAAGGAGAATGAAGAAGCAGGCGAAGATTCAGGAGAGTTCACTGCCCGCTCCTTGATCTTCAGCCACTGCCCTTGTGACTAAAATGGTTCACTACCCTCGTGGAATCCTGACCCCATGTAAATAAAACCGTGACAGCTCATGGGGTGGGAGATATCGCTGTTCCTTAGGACCCTTTTACTAACCCTAATTCGATAGCATATGCTTCCCGTTGGGTAACATATGCTATTGAATTAGGGTTAGTCTGGATAGTATATACTACTACCCGGGAAGCATATGCTACCCGTTTAGGGTTAACAAGGGGGCCTTATAAACACTATTGCTAATGCCCTCTTGAGGGTCCGCTTATCGGTAGCTACACAGGCCCCTCTGATTGACGTTGGTGTAGCCTCCCGTAGTCTTCCTGGGCCCCTGGGAGGTACATGTCCCCCAGCATTGGTGTAAGAGCTTCAGCCAAGAGTTACACATAAAGGCAATGTTGTGTTGCAGTCCACAGACTGCAAAGTCTGCTCCAGGATGAAAGCCACTCAGTGTTGGCAAATGTGCACATCCATTTATAAGGATGTCAACTACAGTCAGAGAACCCCTTTGTGTTTGGTCCCCCCCCGTGTCACATGTGGAACAGGGCCCAGTTGGCAAGTTGTACCAACCAACTGAAGGGATTACATGCACTGCCCCGAATACAAAACAAAAGCGCTCCTCGTACCAGCGAAGAAGGGGCAGAGATGTCGTAGTCAGGTTTAGTTCGTCCGGGGCGGGGATCGATCCTCTAGAGTCGACGAGCTCACTAGTCGCGGCCGCATAACTTCGTATAATGTATGCTATACGAAGTTATCTGCAGTCTCGACAAGCTTGTCGAGAAGTACTAGAGGATCATAATCAGCCATACCACATTTGTAGAGGTTTTACTTGCTTTAAAAAACCTCCCACACCTCCCCCTGAACCTGAAACATAAAATGAATGCAATTGTTGTTGTTAACTTGTTTATTGCAGCTTATAATGGTTACAAATAAAGCAATAGCATCACAAATTTCACAAATAAAGCATTTTTTTCACTGCATTCTAGTTGTGGTTTGTCCAAACTCATCAATGTATCTTATCATGTCTGGATCTGATCACTGCTTGAGCCTAGGAGATCCGAACCAGATAAGTGAAATCTAGTTCCAAACTATTTTGTCATTTTTAATTTTCGTATTAGCTTACGACGCTACACCCAGTTCCCATCTATTTTGTCACTCTTCCCTAAATAATCCTTAAAAACTCCATTTCCACCCCTCCCAGTTCCCAACTATTTTGTCCGCCCACAGCGGGGCATTTTTCTTCCTGTTATGTTTTTAATCAAACATCCTGCCAACTCCATGTGACAAACCGTCATCTTCGGCTACTTT

pFastBacDual backbone: bases 1-4285; 12351-12903

loxP: bases 4286-4319; 12317-12350

CMV promoter: bases 4347-4963; 6751-7367

d2EGFP gene: bases 4973-5818

WPRE: bases 5864-6442

EBNA1 gene: bases 7788-9713

*oriP*: bases 10014-11988
